# Risk prediction of 30-day mortality after stroke using machine learning: a nationwide registry-based cohort study

**DOI:** 10.1186/s12883-022-02722-1

**Published:** 2022-05-27

**Authors:** Wenjuan Wang, Anthony G. Rudd, Yanzhong Wang, Vasa Curcin, Charles D. Wolfe, Niels Peek, Benjamin Bray

**Affiliations:** 1 School of Population Health & Environmental Sciences, Faculty of Life Science and Medicine, King’s College London, London, UK; 2 NIHR Biomedical Research Centre, Guy’s and St Thomas’ NHS Foundation Trust and King’s College London, London, UK; 3 NIHR Applied Research Collaboration (ARC) South London, London, UK; 4 Division of Informatics, Imaging and Data Science, School of Health Sciences, University of Manchester, Manchester, UK; 5 NIHR Manchester Biomedical Research Centre, University of Manchester, Manchester Academic Health Science Centre, Manchester, UK

**Keywords:** Stroke, Machine learning, Statistical analysis, Risk prediction, 30-day mortality, Outcomes, Quality improvement

## Abstract

**Backgrounds:**

We aimed to develop and validate machine learning (ML) models for 30-day stroke mortality for mortality risk stratification and as benchmarking models for quality improvement in stroke care.

**Methods:**

Data from the UK Sentinel Stroke National Audit Program between 2013 to 2019 were used. Models were developed using XGBoost, Logistic Regression (LR), LR with elastic net with/without interaction terms using 80% randomly selected admissions from 2013 to 2018, validated on the 20% remaining admissions, and temporally validated on 2019 admissions. The models were developed with 30 variables. A reference model was developed using LR and 4 variables. Performances of all models was evaluated in terms of discrimination, calibration, reclassification, Brier scores and Decision-curves.

**Results:**

In total, 488,497 stroke patients with a 12.3% 30-day mortality rate were included in the analysis. In 2019 temporal validation set, XGBoost model obtained the lowest Brier score (0.069 (95% CI: 0.068–0.071)) and the highest area under the ROC curve (AUC) (0.895 (95% CI: 0.891–0.900)) which outperformed LR reference model by 0.04 AUC (*p* < 0.001) and LR with elastic net and interaction term model by 0.003 AUC (p < 0.001). All models were perfectly calibrated for low (< 5%) and moderate risk groups (5–15%) and ≈1% underestimation for high-risk groups (> 15%). The XGBoost model reclassified 1648 (8.1%) low-risk cases by the LR reference model as being moderate or high-risk and gained the most net benefit in decision curve analysis.

**Conclusions:**

All models with 30 variables are potentially useful as benchmarking models in stroke-care quality improvement with ML slightly outperforming others.

**Supplementary Information:**

The online version contains supplementary material available at 10.1186/s12883-022-02722-1.

## Introduction

Predicting outcome after stroke can be used at an institutional level to identify whether clinical services are performing below, at or above predicted levels of efficacy [1] which enables remedial action to be taken to support improvement of poorly performing services and to recognise and replicate systems that are delivering better than predicted care.

The complexity of stroke potentially lends itself well to the use of machine learning (ML) algorithms which are able to incorporate large amount of variables and observations into one predictive model [2]. It has been suggested that ML might outperform clinical prediction models based on regression because they make fewer assumptions and can learn complex relationships between predictors and outcomes [3]. However, previous literature has not consistently shown that ML models generate more accurate predictions of stroke outcomes than regression based models, and improvements in methodology and reporting are needed for studies that compare modeling algorithms [4]. Many of the practical machine learning applications are still in their infancy and need to be explored and developed better [5].

There have been numerous ML prediction models developed previously for stroke outcomes [6] but all had some flaws in model building which limited their utility. From a systematic review on predicting outcomes of stroke using machine learning methods, few studies met basic reporting standards for clinical prediction tools and none made their models available in a way which could be used or evaluated [6]. Major improvements in ML study conduct and reporting are needed before it can meaningfully be considered for practice.

This study aimed to use ML and a large, nationally representative dataset containing real-world clinical variables with high potential for practical application to understand if carefully built and reported ML models can provide more accurate predictions of post-stroke mortality than existing methods. The findings of the research are intended to inform the design of predictive analytics used for mortality risk stratification and to support quality improvement in stroke care and benchmark stroke care services.

## Methods

This study is reported according to the TRIPOD guidelines: transparent reporting of a multivariable prediction model for individual prognosis or diagnosis [7].

### Data source

Data were from Sentinel Stroke National Audit Programme (SSNAP), the national registry of stroke care in England, Wales and Northern Ireland. SSNAP is a Healthcare Quality Improvement Partnership (HQIP) register for stroke care quality improvement. Data were collected prospectively, validated by clinical teams and entered into the SSNAP database via a secure web interface. SSNAP is estimated to include an estimated 95% of all adults admitted to hospital with acute stroke (ischaemic or primary intracerebral haemorrhage) in England, Wales and Northern Ireland.

### Study population

The original dataset was collected from 333 teams across England, Wales and Northern Ireland between April 1, 2013 to June 30, 2019. For the generalisation of the model to the population, all patients were included and no specific exclusion criteria for stroke patients.

### Study outcome

The predicted outcome was all-cause 30-day in-hospital mortality post-stroke. All patients had in-hospital status due to the collection procedure in SSNAP. Out-hospital deaths were not available for analysis.

### Variables

In total, 65 variables (Supplementary Table A) were obtained from SSNAP. According to expert advice and literature review, 30 variables collected at arrival and 24 hours were used to build prediction models, including age (band by 5), sex, ethnicity, inpatient at time of stroke, hour of admission, day of week of admission, congestive heart failure, atrial fibrillation (AF), diabetes, hypertension, previous stroke/transient ischaemic attack (TIA), prior anticoagulation if AF, pre-stroke modified Rankin Scale (mRS), National Institutes of Health Stroke Scale (NIHSS) and its 15 components, and type of stroke. Age was obtained from SSNAP as banded by 5 and no continuous age was available for analysis.

### Missing values

Missing data were handled using different methods according to assumptions of missing mechanism after consulting SSNAP team and clinicians. Variables with more than 80% missing were discarded due to high level of missingness. For categorical variables with missing by design/not applicable assumption, missing values were added as a new category. Missing indicator was used for missing by design/not applicable continuous variables. After these, Multiple Imputation with Chained Equations (MICE) [8] was used to impute variables with missing at random assumption for the development dataset. All available variables except for the discarded ones were used in MICE. Five datasets were imputed using MICE and aggregated into one using median. Details for handling missing data were presented in Supplementary Table B. To simulate the future use of the prediction model (i.e. predicting outcomes of individual patients), the validation set and temporal validation set were imputed with the median/mean of each variable.

### Analysis methods

Ordinal categorical variables were coded as integers. Categorical variables that were not ordinal were coded using one hot encoding. Continuous variables remained continuous. Details of coding of variables were presented in Supplementary Table B.

Models were developed/trained using 80% randomly selected data from 2013 to 2018, validated on the remaining 20% data from 2013 to 2018, and temporally validated on data from 2019.

Descriptive statistics were used to compare the characteristic of death/alive at 30-day across the entire datasets, and also used to compare patients’ characteristics for development set, validation set and temporal validation set.

Logistic regression (LR), LR with elastic net [9] with/without interaction terms, and XGBoost [10] were used to build models with the 30 variables. A reference model was developed using the same approach as SSNAP 30-day mortality model [11]: LR with 4 variables (age, NIHSS, previously diagnosed AF, and type of stroke). To make the models comparable, the outcome of LR reference model was in-hospital 30-day mortality.

Best hyperparameters (a parameter that is predefined by the user to control the learning process) were selected on the development set with grid search or random search and cross-validation (CV). Detailed hyperparameter tuning strategy was presented in a repository on Github that was built for this study (https://github.com/SSNAP-Machine-Learning-Project/Post-Stroke_30-day_Mortality_Prediction).

Brier score [12] was used as an overall summative measure of predictive performance. Discrimination was measured by AUC. Calibration was visually assessed with calibration plots [13], and numerically by calibration-in-the-large [13] and calibration slopes [13]. R functions for calculating these measurements were presented in the Github repository. Comparisons of Brier scores and AUCs were conducted with one-way repeated measure Analysis of Variance (ANOVA) [14] on the 500 bootstrap samples. If an overall significant difference had been achieved (significance threshold of *p*-value is 0.05) in one-way repeated measure ANOVA test, post-hoc test [15] was conducted further for pairwise comparison which depicts where exactly the differences occurred. Further, we used DeLong test as an addition method to test the difference between any two methods.

As stroke type is a critical factor for outcomes of stroke patients, we performed a subgroup analysis to investigate the performance of the developed models for patients with different stroke type i.e. infarction and haemorrhage.

Calibration was also evaluated by shift tables, in which we classified patients in the temporal validation set into prespecified categories of low (< 5%), moderate (5–15%), or high risk (> 15%) of 30-day mortality based on two models, creating a 9-way matrix of patients that included risk profiles assigned by the 2 models (low-low, low-moderate, and so on). We then calculated the actual rate of events in these groups and compared them against the observed rates of mortality focusing on discordant categories.

Clinical utility was assessed with decision curve analysis [15] which shows graphically the net benefit obtained by applying the strategy of treating an individual if and only if predicted risk is larger than a threshold in function of the threshold probability. Threshold equals to 0 means treating all since all predicted risk will be larger than 0. Threshold equals to 1 means treating none since all predicted risk will be smaller than 1. All analyses were conducted using R 3.6.2 and occurred between October 2019 to May 2020.

## Results

### Participants

The dataset included information on 488,947 patients (Table 1), of whom 60,362 (12.35%) patients died within 30 days in hospital. The average age group for patients who were dead within 30 days (75–80 year band) was older than the average age group for patients alive (70–75 year band). Patients who died within 30 days had higher prevalence of congestive heart failure (9.4% versus 4.8%), AF (33.0% versus 17.8%), previous stroke/TIA (28.3% versus 26.2%), and a higher proportion of patients with functional impairment pre-stroke (modified Rankin Scale (mRS) mean (SD) 1.78 (1.61) versus 0.95 (1.34)). Patients who died within 30 days were more likely to have intracranial haemorrhage (27.2% versus 9.3%) and have a higher NIHSS (mean (SD) 17.81 (9.34) versus 6.08 (6.14)). Data for all the patients and stratifications by 30-day mortality status were presented in Table 1 with a full list of variables in Supplementary Table C.

**Table 1:** General statistics of cohort with stratification of status at 30 days after hospital admission

	Overall	Alive at 30 days	Dead at 30 days
N	**488,947**	428,585 (87.65%)	60,362 (12.35%)
Age Groups (%)			
*15–60*	76,941 (15.7)	74,238 (17.3)	2703 (4.5)
*61–70*	84,484 (17.3)	79,109 (18.5)	5375 (8.9)
*71–80*	136,728 (28.0)	122,393 (28.6)	14,335 (23.7)
*81+*	190,794 (39.0)	152,845 (35.7)	37,949 (62.9)
Male (%)	249,291 (51.0)	223,849 (52.2)	25,442 (42.1)
Congestive heart failure (%)	26,317 (5.4)	20,656 (4.8)	5661 (9.4)
Hypertension (%)	264,806 (54.2)	232,505 (54.2)	32,301 (53.5)
Atrial fibrillation (%)	96,354 (19.7)	76,417 (17.8)	19,937 (33.0)
Diabetes (%)	102,324 (20.9)	90,104 (21.0)	12,220 (20.2)
Previous stroke or TIA (%)	129,462 (26.5)	112,358 (26.2)	17,104 (28.3)
NIHSS (mean (SD)) (12.9% missing)	7.31 (7.47)	6.08 (6.14)	17.81 (9.34)
Functional impairment pre-stroke (mRS) (mean (SD))	1.05 (1.40)	0.95 (1.34)	1.78 (1.61)
Type of stroke = Haemorrhage (0.7% missing)	55,758 (11.5)	39,472 (9.3)	16,286 (27.2)

*Abbreviation*: *mRS* modified Rankin Scale

### Model specification and performance

In predicting 30-day mortality, 358,588 patients in 2013 to 2018 with 30-day mortality rate of 12.4% were used for developing the model, 89,649 patients in 2013 to 2018 with 30-day mortality rate of 12.2% were used for model validation, and 40,711 patients in 2019 with 30-day mortality rate of 12.3% were used for temporal validation. General characteristics for the development, validation and temporal validation set on all candidate variables are presented in Supplementary Table D. Specifications of the trained models and explanations on how to use them can be found in the repository on Github.

XGBoost obtained the lowest Brier score of 0.069 (95% CI: 0.068 to 0.071) and the highest AUC of 0.895 (95% CI: 0.891 to 0.900) on 2019 temporal validation set (Table 2). The difference between XGBoost and other models were all significant (repeated measure ANOVA and post-hoc test) even though small. On 2019 temporal validation set, XGBoost model performed slightly better than LR with elastic net and interaction terms (AUC difference 0.003, *p* < 0.001) and better than LR and LR with elastic net (AUC difference 0.009, *p* < 0.001). Both Brier score and AUC were improved when adding interaction terms in LR with elastic net and AUC was improved by 0.006 (*p* < 0.001).

**Table 2:** Brier score, AUC, calibration-in-the-large and calibration slope with 95% Confidence Interval (CI) for 2019 temporal validation

Model	Brier score (95% CI)	AUC (95% CI)	Calibration-in-the-large (95% CI)	Calibration slope (95% CI)
LR reference model	0.078 (0.076 to 0.079)	0.854 (0.848–0.860)	0.017 (− 0.033–0.066)	0.977 (0.952–0.998)
LR	0.073 (0.071–0.074)	0.886 (0.881–0.891)	0.200 (0.145–0.257)	1.055 (1.028–1.081)
LR with elastic net	0.073 (0.071–0.074)	0.886 (0.882–0.891)	0.212 (0.158–0.265)	1.075 (1.050–1.098)
LR with elastic net and interaction terms	0.071 (0.069–0.073)	0.892 (0.887–0.897)	0.305 (0.252–0.356)	1.116 (1.090–1.144)
XGBoost	0.069 (0.068–0.071)	0.895 (0.891–0.900)	0.142 (0.090–0.190)	1.077 (1.050–1.102)

Models with 30 variables outperformed LR reference model with 4 variables (Table 2) with both Brier score and AUC. With 30 variables, the prediction accuracy was improved by 0.041 AUC (*p* < 0.001) for 2019 temporal validation. Results for validation were presented in Supplementary Table E.

**Fig. 1:**
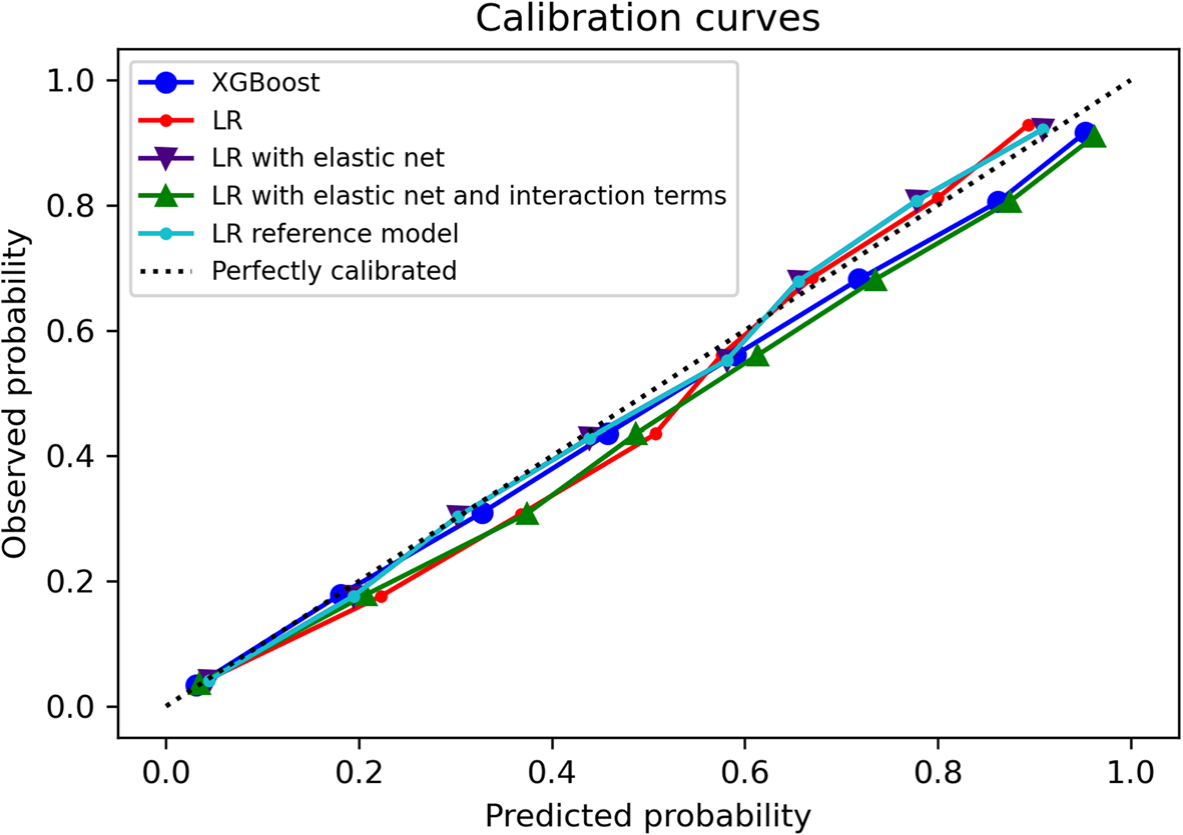
Calibration plots of all models on 2019 temporal validation set

Calibration-in-the-large (Table 2) were slightly higher than 0 for all models indicating underestimated average predicted risk except for LR reference model. LR reference model had a calibration slope (Table 2) smaller than 1 suggesting that the estimated risks were too extreme whilst calibration slope for other models was slightly larger than 1 suggesting that the estimated risks were too moderate. All calibration curves (Fig. 1 on 2019 validation set and Supplementary Fig. A on validation set) were close to the diagonal for low (< 5%) and moderate (5–15%) risk groups but slightly above the diagonal line (≈1%) for high-risk (> 15%) groups which indicated underestimation [16].

Notably, 1648 (8.1%) low-risk cases by LR reference model were more appropriately reclassified as being moderate or high risk by XGBoost (Table 3), similarly, 1328 (6.3%) cases by LR model, 1429 (6.7%) by LR with elastic net, 1379 (6.4%) by LR with elastic net and interaction terms (Supplementary Table F).

**Table 3:** Shift table of reclassification with different models on 2019 temporal validation set

	Low risk (< 5%)		Moderate risk (5–15%)		High risk (> 15%)		All	
	Number (% in Low risk)	observed mortality (%)	Number (% in Moderate risk)	observed mortality (%)	Number (% in High risk)	observed mortality (%)	Number (% in All)	observed mortality (%)
	**LR reference model (4 variables)**							
XGBoost								
Number in Low risk	19,664 (92.3)	1.45	3549 (32.4)	3.07	6 (0.07)	33.33	23,219 (57.0)	1.71
Number in Moderate risk	1351 (6.7)	8.66	5942 (54.3)	8.16	1007 (11.9)	10.43	8300 (20.4)	8.52
Number in High risk	297 (1.4)	51.52	1455 (12.3)	28.73	7440 (88.0)	44.96	9192 (22.6)	42.60
All	21,312 (100)	2.61	10,946 (100)	9.25	8453 (100)	40.84	40,711 (100)	12.33
	**LR**							
LR with elastic net and interaction terms								
Number in Low risk	22,269 (96.9)	1.75	1286 (13.3)	3.65	0 (0)	0	23,555 (57.9)	1.85
Number in Moderate risk	705 (3.1)	6.38	7727 (79.8)	9.63	309 (3.8)	10.36	8741 (21.5)	9.39
Number in High risk	0 (0)	0	669 (6.9)	24.51	7746 (96.2)	46.46	8415 (20.7)	44.72
All	22,974 (100)	1.18	9682 (100)	9.50	22,974 (100)	44.85	40,711 (100)	12.33
	**LR with elastic net and interaction terms**							
XGBoost								
Number in Low risk	22,707 (96.4)	1.65	512 (5.9)	4.49	0 (0)	0	23,219 (57.0)	1.71
Number in Moderate risk	846 (3.6)	7.33	7359 (84.2)	8.59	95 (1.1)	13.68	8300 (20.4)	8.52
Number in High risk	2 (0)	0	870 (10)	19.08	8320 (98.9)	45.07	9192 (22.6)	42.60
All	23,555 (100)	1.85	8741 (100)	9.39	8415 (100)	44.72	40,711 (100)	12.33

Compared to LR model (Table 3), LR with elastic net and interaction terms more appropriately reclassified 705 (3.1%) low-risk cases by LR model to be at moderate-risk. 1286 (13.3%) moderate-risk cases by LR model were appropriately reclassified as at low-risk by LR with elastic net and interaction terms. LR with elastic net and LR had very small difference in the risk groups (Supplementary Table G).

Compared to LR with elastic net and interaction terms (Table 3), XGBoost more appropriately reclassified 846 (3.6%) low-risk cases by LR with elastic net and interaction terms to be at moderate risk. 512 (5.9%) moderate risk by LR with elastic net and interaction terms were more appropriately reclassified to be at low risk. 870 (10%) moderate-risk cases by LR with elastic net and interaction terms were more appropriately reclassified to be at high risk.

**Fig. 2:**
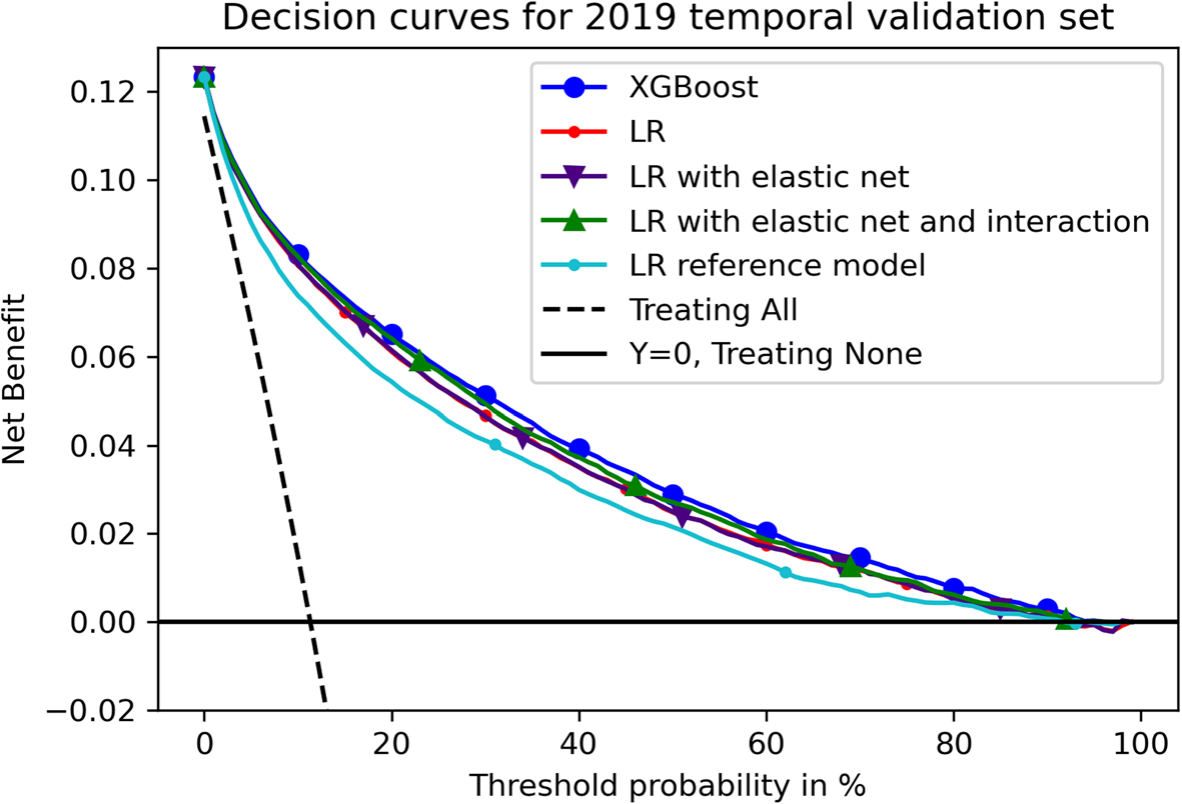
Decision curves of all models on 2019 temporal validation set

From the decision curves on 2019 temporal validation set (Fig. 2), all models gained net benefits [17] with the risk threshold between 5 to 90% compared to treating all and treating none whilst among all models, Xgboost performed the best. When the threshold was between 5 to 10%, the decision curve overlapped between XGBoost and LR related models whilst they were all better than LR reference model. Decision curves on validation set are in Supplementary Fig. B which had similar results.

**Fig. 3:**
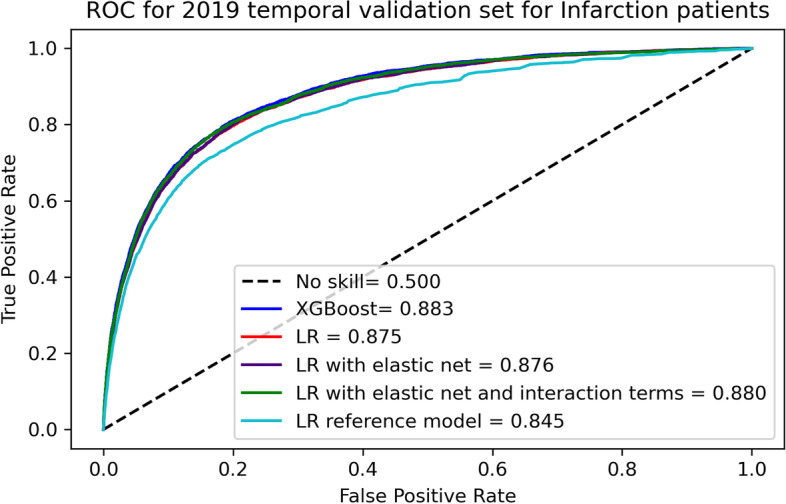
AUC-ROC for infarction patients in 2019 temporal validation set

**Fig. 4:**
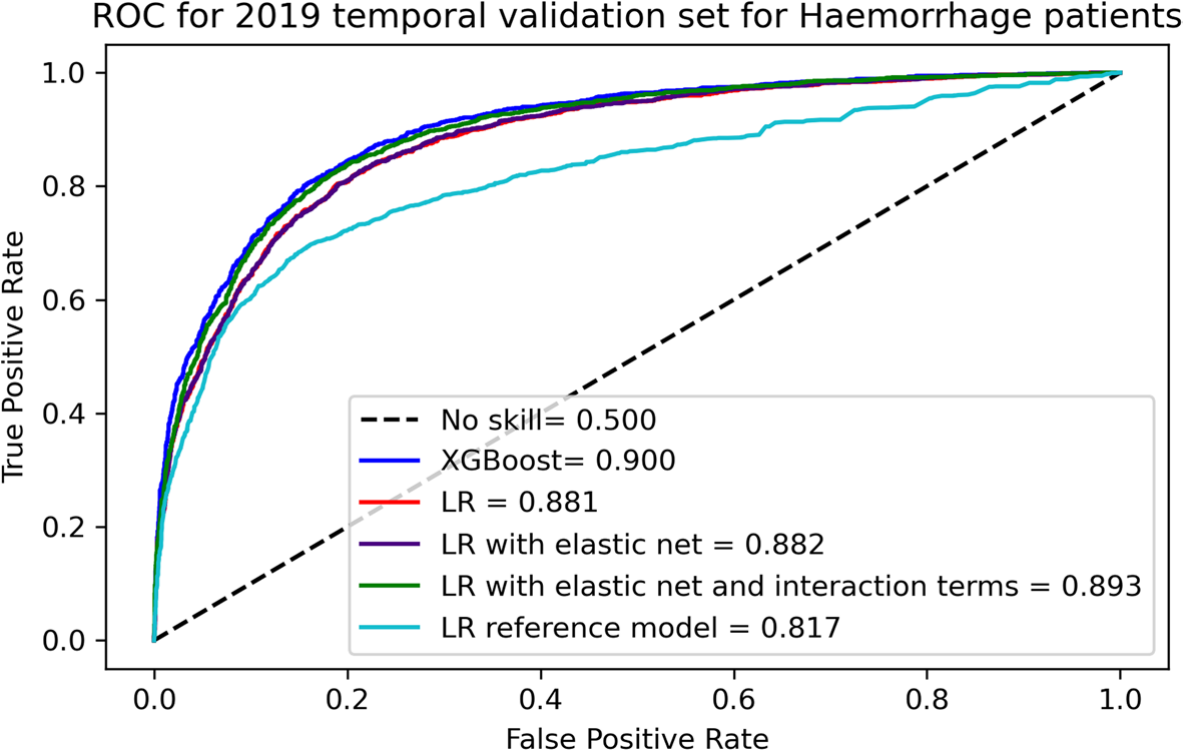
AUC-ROC for haemorrhage patients in 2019 temporal validation set

For different stroke types, XGBoost and other LR models performed slightly better on patients with haemorrhage (AUC 0.900 [95% CI: 0.890–0.909] for XGBoost) than infarction (AUC: 0.833 [95% CI: 0.878–0.889]) but LR reference model with 4 variables performed better for patients with infarction (AUC: 0.845 [95% CI: 0.837–0.851]) rather than patients with haemorrhage (AUC: 0.817 [95% CI: 0.803–0.831]) (Figs. 3 and 4). Calibration curves showed that all models in the 2019 temporal validation set had almost perfect calibration for haemorrhage patients (Supplementary Fig. C) but XGBoost underestimated the risk for very high risk of infarction (Supplementary Fig. D). Decision curves showed that for both stroke types XGBoost had the highest net benefit on 2019 temporal validation set (Supplementary Fig. E and F).

According to the feature importance calculated from XGBoost model, NIHSS at arrival, level of consciousness, age, type of stroke and pre-sroke mRS were the most important features in making the predictions (see the first 20 most important features in Supplementary Fig. G). Compared to the four variables used in LR reference model, AF was less important than presroke mRS at predicting the 30-day mortality.

## Discussion

In this study we explored the performance of XGBoost and LR related models for predicting risk of 30-day mortality after stroke. Our findings showed that the improvement of XGBoost was modest compared to LR with elastic net and interaction terms (AUC difference 0.003, *p* < 0.001) and larger compared to LR and LR with elastic net (AUC difference 0.009, *p* < 0.001). There has been mixed signals about whether ML outperforms LR models [6]. In our case, with nearly a half million patients, the gain of ML was small compared to LR with elastic net and interaction terms even though significant due to the large dataset. Also, previous studies used simpler LR models (e.g. LR or least absolute shrinkage and selection operator (LASSO)) with no interactions which constrains the models to learn only linear relationships whilst ML models are capable of learning very complicated relationships. XGBoost and LR with elastic net and interaction terms performed better than LR showed that there is an opportunity to improve the accuracy by incorporating interactions between risk predictors.

When including more variables in the model, AUCs were improved by 0.041 (*p* < 0.001) for both LR related models and XGBoost which showed the potential of improving the accuracy by using more variables combining data-driven variable selection (i.e. LR with elastic net achieves variable selection by shrinking the coefficients of the variables and XGBoost by calculating variable importance).

A variety of models have been developed previously to predict post-stroke mortality, including ML models [6], LR models and scores [18]. The model developed by Bray et al. [11] has been externally validated twice [19, 20] with different population. Our reference model was developed with the same approach but with more patients enrolled between 2013 and 2019 compared to Bray et al. model [11] which used patients from 2013. The AUC with LR reference model was slightly lower (0.854 (0.848–0.860) versus 0.86 (0.85–0.88)) with the Bray et al. model [11]. Our model with 30 variables using XGBoost had a higher AUC of 0.896 (0.891–0.900).

Existing scores for 30-day mortality prediction are PLAN [21] and IScore [22]. PLAN was externally validated with AUC (0.87 (0.85–0.90)) [23] and IScore with AUC (0.88 (0.86–0.91)) which were higher compared to the original studies (PLAN: AUC 0.79, IScore: AUC 0.79–0.85). Due to lack of certain variables (PLAN score: cancer, Iscore: Cancer, renal dialysis, stroke scale score, non-lacunar, and abnormal glucose level), we could not externally validate these scores.

The subgroup analysis with types of stroke showed that the models with 30 variables predicted better at haemorrhage patients than infarction patients. The reasons for this are not clear, but might relate to the proportionally higher number of mortality events in the patients with haemorrhagic stroke. The feature importance of the variables in the XGBoost model were consistent with previous literature about prognostic factors after stroke, with NIHSS, age, stroke type and prestroke mRS being the most important predictors. Notably, many individual components of the NIHSS score also contributed to the predictions in addition to the overall NIHSS, indicating that there is value in using all components of the NIHSS as part of prognostic models.

ML models have been notably performing well with non-structured data such as text mining and imaging. However, in terms of structured data, the advantages of LR models in interpretability outweigh the small improvement of the prediction accuracy by ML models. Furthermore, even though the use of ML for predicting stroke outcomes is increasing, few met basic reporting standards for clinical prediction tools and none made their models available in a way which could be used or evaluated [6]. Major issues found in the ML studies are small dataset size, lack of calibration and external validation, lack of details in hyperparameter tuning, lack of decision curve analysis, and the non-availability of the final model [6]. None of the ML model were externally validated due to the low quality of reporting and lack of final model. From the low reporting quality, few external validation of ML models, and lack of guidelines on developing and reporting, ML still has a long way to go in being accepted by clinicians and implemented in real-world setting.

### Strengths and limitations

This is by far the largest stroke dataset used for developing prediction of post-stroke mortality model using ML (around 0.5 million versus < 1000 in previous ML post-stroke mortality prognosis studies [6] and 77,653 as the largest, to the best of our knowledge, for LR model/score-based approach [24]). The participants in the study are presentative for the nation and complete in terms of a nearly complete national population of hospitalised stroke and richness of available data and data quality.

The models were built with robust approaches and reported according to the TRIPOD reporting guidelines with several terms adjusted for ML studies such as hyperparameter turning and final model presentation. For missing data, we explored the missing mechanism which was not explored in previous studies before imputing or fitting the model [18]. Hyperparameter selection was well reported in our study but not in previous ML studies [6]. Temporal validation was performed to make sure that the models can apply to data collected after the model was developed. Finally, a repository on Github was built to share the pre-trained models for other studies to externally validate.

The main limitations are that we were restricted to using variables available in SSNAP and there may be other variables (e.g. imaging) that might improve the accuracy of prediction. We used 30 variables that were collected within SSNAP, which might not be available in other databases. However, the variables are generally available in stroke registries which can benefit from the models developed in this study. The validation was limited to temporal validation and ideally the model should be validated in external data from other data sources. Finally, death outcomes were limited to inpatient mortality and it was not possible to ascertain deaths occurring outside hospital within 30 days.

## Conclusions

The potential gain for machine learning versus carefully developed statistical models to produce more accurate predictive analytics using stroke registries is likely to be modest. Compared to the reference model with 4 variables, all models with 30 variables are potentially useful as benchmarking models in quality improvement of stroke care with ML slightly outperforming others. These findings emphasise the usefulness of collecting more detailed clinical data to support predictive analytics for quality improvement in stroke care.

## Supplementary Information

**Additional file 1.**

## Data Availability

The data that support the findings of this study are available from SSNAP (www.strokeaudit.org) but restrictions apply to the availability of these data, which were used under license for the current study, and so are not publicly available. Data are however available from the SSNAP upon reasonable request and with permission of HQIP. Because of the sensitive nature of the data collected for this study, requests to access the dataset from qualified researchers trained in human subject confidentiality protocols may be sent to the HQIP at https://www.hqip.org.uk/national-programmes/accessing-ncapop-data/.

## Abbreviations

ML: Machine learning
LR: Logistic regression
AUC: Area under the ROC curve
SSNAP: Sentinel Stroke National Audit Programme
HQIP: Healthcare Quality Improvement Partnership
MICE: Multiple Imputation with Chained Eqs
CV: Cross-validation
ANOVA: Analysis of Variance
