# Correction: Risk prediction of 30-day mortality after stroke using machine learning: a nationwide registry-based cohort study

**DOI:** 10.1186/s12883-022-02840-w

**Published:** 2022-08-25

**Authors:** Wenjuan Wang, Anthony G. Rudd, Yanzhong Wang, Vasa Curcin, Charles D. Wolfe, Niels Peek, Benjamin Bray

**Affiliations:** 1 School of Population Health & Environmental Sciences, Faculty of Life Science and Medicine, King’s College London, London, UK; 2 NIHR Biomedical Research Centre, Guy’s and St Thomas’ NHS Foundation Trust and King’s College London, London, UK; 3 NIHR Applied Research Collaboration (ARC) South London, London, UK; 4 Division of Informatics, Imaging and Data Science, School of Health Sciences, University of Manchester, Manchester, UK; 5 NIHR Manchester Biomedical Research Centre, University of Manchester, Manchester Academic Health Science Centre, Manchester, UK

**Correction: BMC Neurol 22, 195 (2022)**

**https://doi.org/10.1186/s12883-022-02722-1**

Following publication of the original article [1], the authors reported an error in Fig. 1. The same error was made in Figure A, C and D in supplemental material. The context was not affected as the error was made when replotting during the revision. We apologise for the error.

The correct Fig. 1 and supplemental material are provided below.

**Fig. 1:**
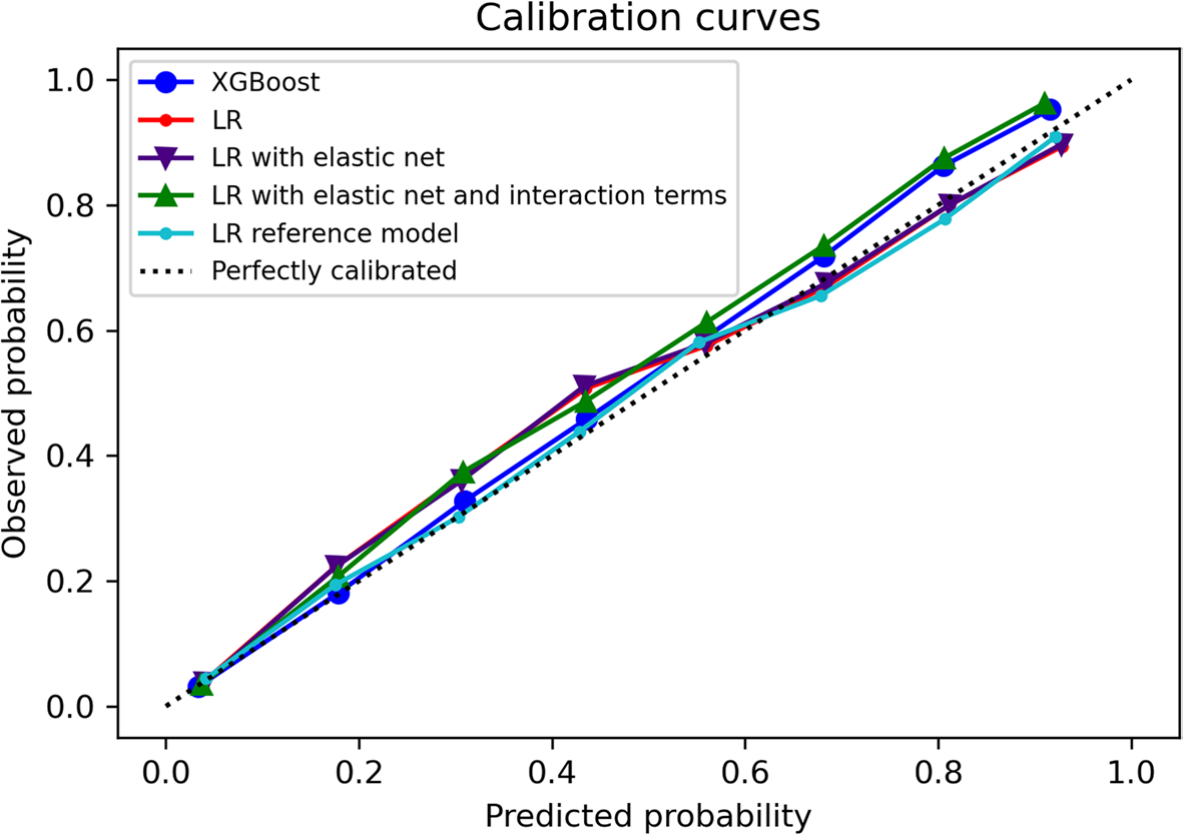
Calibration plots of all models on 2019 temporal validation set

The original article [1] has been updated.

## Supplementary Information

**Additional file 1.**
