# Supplementary material for: Correction: Risk prediction of 30-day mortality after stroke using machine learning: a nationwide registry-based cohort study
Source: BMC Neurol. 2022 Aug 25;22:319. doi: 10.1186/s12883-022-02840-w (PMC9404635; doi:10.1186/s12883-022-02840-w)
Supplement: Supplementary file 1 — Additional file 1. [file 12883_2022_2840_MOESM1_ESM.docx]

**SUPPLEMENTAL MATERIAL**

Table A: All variables in the dataset, source of variables, role and use of variables

| All Variables | Source in SSNAP Dataset (SSNAP data collection sheet is attached) | Patient characteristics, clinical presentation, intervention and outcomes | Role in this study/how it was used | Outcomes predicted using the predictors |
| --- | --- | --- | --- | --- |
| Demographics/Onset/Arrival |  |  |  |  |
| Patient ID |  | - | merging records |  |
| Team number | Derived uniquely for this project to reduce the probability of reidentification | - | Discard – irrelevant |  |
| Sex | 1.6 | patient characteristics | Predictor and Multiple imputation | 30-day mortality |
| Ethnicity | Derived [White, Mixed/Multiple ethnic groups, Asian/Asian British, Black/ African/Caribbean/Black British, Other] | patient characteristics | Predictor and Multiple imputation | 30-day mortality |
| age | Age band by 5 from 15 to 125 to reduce probability of reidenfication | patient characteristics | Predictor and Multiple imputation | 30-day mortality |
| Inpatient at time of stroke | 1.1 | clinical presentation | Predictor and Multiple imputation | 30-day mortality |
| Hour of admission | Derived in time bands to reduce probability of reidentification. | patient characteristics | Predictor and Multiple imputation | 30-day mortality |
| Day of week of admission | Derived from date to reduce probability of reidientification | patient characteristics | Predictor and Multiple imputation | 30-day mortality |
| Year of Admission | Derived from date to reduce probability of reidientification | patient characteristics | Multiple imputation |  |
| Time from onset to admission (minutes) | Derived | patient characteristics | Predictor and Multiple imputation | Clinician comment: Difficult; probably omit. Short time to admission could be either a predictor of lower mortality because treatment given quickly but confounded by severe strokes presenting quicker |
| Direct admission to a stroke unit within 4 hours of clock start | Derived | Intervention | Predictor and Multiple imputation |  |
| Case mix/First 24 hours |  |  |  |  |
| Congestive heart failure | 2.1.1 | Patient characteristic | Predictor and Multiple imputation | 30-day mortality |
| hypertension | 2.1.2 | Patient characteristic | Predictor and Multiple imputation | 30-day mortality |
| Atrial fibrillation (AF) | 2.1.3 | Patient characteristic | Predictor and Multiple imputation | 30-day mortality |
| diabetes | 2.1.4 | Patient characteristic | Predictor and Multiple imputation | 30-day mortality |
| Previous stroke/tia | 2.1.5 | Patient characteristic | Predictor and Multiple imputation | 30-day mortality |
| Prior anticoagulation if AF^*^ | 2.1.7 | Patient characteristic | Predictor and Multiple imputation | 30-day mortality |
| International normalized ratio (INR) at admission | 2.1.7.b (if 2.1.7) | clinical presentation | Discard – too much missing values |  |
| Modified Rankin Scale pre stroke | 2.2 | Patient characteristic | Predictor and Multiple imputation | 30-day mortality |
| NIHSS at arrival | 2.3 | clinical presentation | Predictor and Multiple imputation | 30-day mortality |
| level of consciousness | 2.3.1 |  | Predictor and Multiple imputation | 30-day mortality |
| answers questions | 2.3.2 |  | Predictor and Multiple imputation | 30-day mortality |
| obeys commands | 2.3.3 |  | Predictor and Multiple imputation | 30-day mortality |
| best gaze | 2.3.4 |  | Predictor and Multiple imputation | 30-day mortality |
| visual deficits | 2.3.5 |  | Predictor and Multiple imputation | 30-day mortality |
| facial weakness | 2.3.6 |  | Predictor and Multiple imputation | 30-day mortality |
| left arm weakness | 2.3.7 |  | Predictor and Multiple imputation | 30-day mortality |
| right arm weakness | 2.3.8 |  | Predictor and Multiple imputation | 30-day mortality |
| left leg weakness | 2.3.9 |  | Predictor and Multiple imputation | 30-day mortality |
| right leg weakness | 2.3.10 |  | Predictor and Multiple imputation | 30-day mortality |
| ataxia | 2.3.11 |  | Predictor and Multiple imputation | 30-day mortality |
| sensory loss | 2.3.12 |  | Predictor and Multiple imputation | 30-day mortality |
| best language | 2.3.13 |  | Predictor and Multiple imputation | 30-day mortality |
| dysarthria | 2.3.14 |  | Predictor and Multiple imputation | 30-day mortality |
| extinction | 2.3.15 |  | Predictor and Multiple imputation | 30-day mortality |
| Type of stroke | 2.5 | clinical presentation | Predictor and Multiple imputation | 30-day mortality |
| Received thrombolysis | 2.6 Only valid for ischaemic stroke | intervention | Multiple imputation |  |
| Thrombolysed within 1h | Derived Only valid if thrombolysed | intervention | Multiple imputation |  |
| Time from clock start to thrombolysis | Derived Only valid if thrombolysed | intervention | Multiple imputation |  |
| Swallow screen within 4 hours if applicable | 2.10 | Intervention | Multiple imputation |  |
| Intra-arterial intervention for acute stroke | 2.11 | intervention | Discard – too much missing values |  |
| Systolic blood pressure on arrival | 2.12 collected from Dec 2017 | clinical presentation | Discard – too much missing values |  |
| Time from clock start to brain scan (minutes) | Derived | intervention | Multiple imputation |  |
| Assessments-First 72 hours/by discharge |  |  |  |  |
| Palliative care within 72 hours | 3.1 | Prognosis | Multiple imputation |  |
| Assessment by a stroke specialist nurse | 3.2 | intervention | Multiple imputation |  |
| Time from clock start to assessment by a stroke specialist nurse | Derived | intervention | Discard – irrelevant |  |
| Assessment by a stroke specialist physician within 72 hours of clock start | 3.3a | intervention | Multiple imputation |  |
| Time between clock start and stroke specialist physician review | Derived | intervention | Discard – irrelevant |  |
| Formal swallow assessment within 72 hours of clock start if applicable | 3.4 | intervention | Multiple imputation |  |
| Required occupational therapy | 3.5 | Intervention | Multiple imputation |  |
| Assessment by an occupational therapist within 72 hours of clock start if applicable | 3.5 | intervention | Multiple imputation |  |
| Percentage of days occupational therapy received | Derived | intervention | Multiple imputation |  |
| Required physiotherapy | 3.6 | intervention | Multiple imputation |  |
| Assessment by an physiotherapist within 72 hours of clock start if applicable | 3.6 | intervention | Multiple imputation |  |
| Percentage of days physiotherapy received | Derived | intervention | Multiple imputation |  |
| Required speech and language therapy | 3.7 | intervention | Multiple imputation |  |
| Assessment by a speech and language therapist within 72 hours of clock start if applicable | 3.7 | intervention | Multiple imputation |  |
| Percentage of days speech and language therapy received | Derived | intervention | Multiple imputation |  |
| Patient condition in first 7 days |  |  |  |  |
| Worst level of consciousness | 5.1 | Affected by treatments | Multiple imputation |  |
| pneumonia requiring antibiotics | 5.3 | Try to avoid by swallow assessments | Multiple imputation |  |
| Discharge/Transfer |  |  |  |  |
| Discharge destination | 7.1 | Outcome | Extract outcome/ Multiple imputation |  |
| Modified Rankin Scale at discharge/transfer | 7.4 | Outcome | Discard |  |
| If discharged to a care home, previously a resident or not | 7.5 | Outcome | Multiple imputation |  |
| Discharged to an early supported discharge team | 7.7 | Outcome | Multiple imputation |  |
| Help with activities of daily living | 7.9 | Outcome | Multiple imputation |  |
| Atrial fibrillation on discharge | 7.10 | Outcome | Multiple imputation |  |
| At least 90% of inpatient stay on the stroke unit | Derived | Outcome | Multiple imputation |  |
| Total inpatient length of stay | Derived | Outcome | Multiple imputation |  |
| Six months follow-up |  |  |  |  |
| Modified Rankin Scale Score at six months | 8.4 | Outcome | Discard – too many missing values |  |

Table B: Summary of missing values for each variable, handling method and coding method.

| Variables | Missing values | Missing values percentage | Method for handling missing data | Coding |
| --- | --- | --- | --- | --- |
| 30-day Mortality | No | - | - | 0-No, 1-Yes |
| Variables used in model building |  |  |  |  |
| Sex | No | - | - | 0-Female, 1-Male |
| Ethnicity | No | - | - | Asian, Black, Mixed, Other, Unknown, White: One hot encoding |
| age | No | - | - | Band by 5 from age 15 to age 125, levels: 0-20 |
| Inpatient at time of stroke | No | - | - | 0-No, 1-Yes |
| Hour of admission | No | - | - | 6 Levels, 4 hours band: One hot encoding |
| Day of week of admission | No | - | - | Monday – Sunday: One hot encoding |
| Year of Admission | No | - | - | 2013-2019: One hot encoding |
| Time from onset to admission | Yes | 37.28% | Indicator function | Continuous |
| Congestive heart failure | No | - | - | 0-No, 1-Yes |
| hypertension | No | - | - | 0-No, 1-Yes |
| Atrial fibrillation | No | - | - | 0-No, 1-Yes |
| diabetes | No | - | - | 0-No, 1-Yes |
| Prior anticoagulation if AF | Yes | 58.86% | New category | No, No but,  Unknown, Yes: One hot encoding |
| Previous stroke tia |  |  |  |  |
| Rankin scale prestroke | No | - | - | 0-5 |
| NIHSS at arrival | No^*^ (but not accurate) |  | Sum of imputed NIHSS subcomponents | 0-42 |
| level of consciousness | No | - | - | 0-3 |
| answers questions | Yes | 8.26% | Multiple imputation | 0-2 |
| obeys commands | Yes | 8.15% | Multiple imputation | 0-2 |
| best gaze | Yes | 9.03% | Multiple imputation | 0-2 |
| visual deficits | Yes | 9.96% | Multiple imputation | 0-3 |
| facial weakness | Yes | 8.19% | Multiple imputation | 0-3 |
| left arm weakness | Yes | 8.10% | Multiple imputation | 0-4 |
| right arm weakness | Yes | 8.10% | Multiple imputation | 0-4 |
| left leg weakness | Yes | 8.26% | Multiple imputation | 0-4 |
| right leg weakness | Yes | 8.27% | Multiple imputation | 0-4 |
| ataxia | Yes | 10.5% | Multiple imputation | 0-2 |
| sensory loss | Yes | 9.95% | Multiple imputation | 0-2 |
| best language | Yes | 8.41% | Multiple imputation | 0-3 |
| dysarthria | Yes | 9.00% | Multiple imputation | 0-2 |
| extinction | Yes | 9.31% | Multiple imputation | 0-2 |
| Type of stroke | Is unknown missing values? | 0.7% | Unknown as a new category | Infarction, Primary Intracerebral Haemorrhage, Unknown: One hot encoding |
| Worst level of consciousness | No | - | - | 0-3 |
| pneumonia requiring antibiotics |  |  | Unknown as a new category | No, Not known, Yes: One hot encoding |
| Variables used in multiple imputation |  |  |  |  |
| Received thrombolysis | No | - | - | No, No but, Yes: One hot encoding |
| thrombolysed_within_1h | Yes | 11.50% (conditional to Received thrombolysis) | New category | No, NoA, Yes: One hot encoding |
| Time from clock start to thrombolysis | Yes | 11.50% (conditional to Received thrombolysis) | Indicator function | Continuous |
| Palliative care within 72 hours | No | - | - | 0-No, 1-Yes |
| Discharge destination (combined with “if discharged to a care home, previously a care home resident or not) | No | - | - | 7 Levels: Died Was discharged home Was discharged to a care home No ... Was transferred to ESD/CRT NOT PARTICIPATING: One hot encoding |
| Modified Rankin Scale at discharge/transfer | No | - | - | 0-6 |
| If discharged to a care home, previously a resident or not | No | - | - | Combined with discharge destination |
| Help with activities of daily living | Yes | 14.55% | All missing values are patients who died: New category | Died, No, Yes: One hot encoding |
| Atrial fibrillation on discharge | Yes | 14.55% | All missing values are patients who died: New category | Died, No, Yes: One hot encoding |
| Time from clock start to brain scan | Yes | 0.70% | Indicator function | Continuous |
| Direct admission to a stroke unit within 4 hours of clock start | Yes | 5.54% | New category | No, Unknown, Yes: One hot encoding |
| At least 90% of inpatient stay on the stroke unit | No | - | - | 0-No, 1-Yes |
| Assessment by a stroke specialist physician within 24 hours of clock start | No | - | - | 0-No, 1-Yes |
| Assessment by a stroke specialist nurse | No | - | - | 0-No, 1-Yes |
| Swallow screen within 4 hours if applicable | Yes | 8.88% | New category | No, Unknown, Yes: One hot encoding |
| Formal swallow assessment within 72 hours of clock start if applicable | Yes | 61.26% | New category | No, Unknown, Yes: One hot encoding |
| Required occupational therapy | No | - | - | 0-No, 1-Yes |
| Percentage of days occupational therapy received | Yes | 32.67% | Impute as 0 | Continuous |
| Assessment by an occupational therapist within 72 hours of clock start if applicable | Yes | 13.98% | New category | No, Unknown, Yes: One hot encoding |
| Required physiotherapy | No | - | - | 0-No, 1-Yes |
| Percentage of days physical therapy received | Yes | 31.84% | Impute as 0 | Continuous |
| Assessment by an physicaltherapist within 72 hours of clock start if applicable | Yes | 11.12% | New category | No, Unknown, Yes: One hot encoding |
| Required speech and language therapy | No | - | - | 0-No, 1-Yes |
| Percentage of days speech and language therapy received | Yes | 60.94% | Impute as 0 | Continuous |
| Assessment by a speech and language therapist within 72 hours of clock start if applicable | Yes | 52.56% | New category | No, Unknown, Yes: One hot encoding |
| Discharged to an early supported discharge team | Yes | 14.55% | All missing values are patients who died | Died, No, Yes non-specialist, Yes stroke/neurology specific: One hot encoding |
| Discarded variables |  |  |  |  |
| Patient ID | No | - | - | - |
| Team number | No | - | - | - |
| INR at admission | Yes | 98.76% | - | - |
| Systolic blood pressure on arrival | Yes | 96.86% | - | - |
| Intra-arterial intervention for acute stroke | Yes | 44.31% | - | - |
| Time between clock start and stroke specialist physician review | Yes | 6.1% | - | - |
| Time from clock start to assessment by a stroke specialist nurse | Yes | 5.46% | - | - |
| Modified Rankin Scale Score at six months | Yes | 76.90% | - | - |

**Table C: Patient characteristics,** stratified by death status within 30 days

|  | 30-day mortality | | |
| --- | --- | --- | --- |
| Outcome |  | Alive | Died |
| n | 488947 | 428585 (87.65%) | 60362 (12.35%) |
| Age_Groups_by5 (%) |  |  |  |
| (15,20] | 494 (0.1) | 466 (0.1) | 28 (0.0) |
| (20,25] | 753 (0.2) | 733 (0.2) | 20 (0.0) |
| (25,30] | 1282 (0.3) | 1257 (0.3) | 25 (0.0) |
| (30,35] | 2144 (0.4) | 2097 (0.5) | 47 (0.1) |
| (35,40] | 3676 (0.8) | 3590 (0.8) | 86 (0.1) |
| (40,45] | 7384 (1.5) | 7171 (1.7) | 213 (0.4) |
| (45,50] | 13633 (2.8) | 13203 (3.1) | 430 (0.7) |
| (50,55] | 20678 (4.2) | 20000 (4.7) | 678 (1.1) |
| (55,60] | 26897 (5.5) | 25721 (6.0) | 1176 (1.9) |
| (60,65] | 35452 (7.3) | 33504 (7.8) | 1948 (3.2) |
| (65,70] | 49032 (10.0) | 45605 (10.6) | 3427 (5.7) |
| (70,75] | 61833 (12.6) | 56244 (13.1) | 5589 (9.3) |
| (75,80] | 74895 (15.3) | 66149 (15.4) | 8746 (14.5) |
| (80,85] | 81513 (16.7) | 68830 (16.1) | 12683 (21.0) |
| (85,90] | 66069 (13.5) | 52566 (12.3) | 13503 (22.4) |
| (90,95] | 34526 (7.1) | 25557 (6.0) | 8969 (14.9) |
| (95,100] | 7890 (1.6) | 5354 (1.2) | 2536 (4.2) |
| (100,105] | 758 (0.2) | 509 (0.1) | 249 (0.4) |
| (105,110] | 15 (0.0) | 8 (0.0) | 7 (0.0) |
| (110,115] | 9 (0.0) | 9 (0.0) | 0 (0.0) |
| (115,120] | 14 (0.0) | 12 (0.0) | 2 (0.0) |
| Male (%) | 249291 (51.0) | 223849 (52.2) | 25442 (42.1) |
| Ethnicity (%) |  |  |  |
| White | 432911 (88.5) | 377906 (88.2) | 55005 (91.1) |
| Black | 6476 (1.3) | 6060 (1.4) | 416 (0.7) |
| Asian | 14547 (3.0) | 13378 (3.1) | 1169 (1.9) |
| Mixed | 1815 (0.4) | 1659 (0.4) | 156 (0.3) |
| Other | 5745 (1.2) | 5248 (1.2) | 497 (0.8) |
| Unknown | 27453 (5.6) | 24334 (5.7) | 3119 (5.2) |
| Inpatient_at_time_of_stroke (%) | 27100 (5.5) | 20879 (4.9) | 6221 (10.3) |
| hour_of_admission_4h_band (%) |  |  |  |
| 00:00:00 to 03:59:59 | 25117 (5.1) | 21726 (5.1) | 3391 (5.6) |
| 04:00:00 to 07:59:59 | 26833 (5.5) | 22546 (5.3) | 4287 (7.1) |
| 08:00:00 to 11:59:59 | 133886 (27.4) | 116202 (27.1) | 17684 (29.3) |
| 12:00:00 to 15:59:59 | 136361 (27.9) | 121551 (28.4) | 14810 (24.5) |
| 16:00:00 to 19:59:59 | 102265 (20.9) | 90690 (21.2) | 11575 (19.2) |
| 20:00:00 to 23:59:59 | 64485 (13.2) | 55870 (13.0) | 8615 (14.3) |
| day_of_week_of_admission (%) |  |  |  |
| Monday | 76508 (15.6) | 67453 (15.7) | 9055 (15.0) |
| Tuesday | 73072 (14.9) | 64201 (15.0) | 8871 (14.7) |
| Wednesday | 72527 (14.8) | 63667 (14.9) | 8860 (14.7) |
| Thursday | 71484 (14.6) | 62740 (14.6) | 8744 (14.5) |
| Friday | 70940 (14.5) | 62501 (14.6) | 8439 (14.0) |
| Saturday | 61969 (12.7) | 53904 (12.6) | 8065 (13.4) |
| Sunday | 62447 (12.8) | 54119 (12.6) | 8328 (13.8) |
| year_of_admission (%) |  |  |  |
| 2013 | 50282 (10.3) | 44279 (10.3) | 6003 (9.9) |
| 2014 | 76350 (15.6) | 66873 (15.6) | 9477 (15.7) |
| 2015 | 77977 (15.9) | 68035 (15.9) | 9942 (16.5) |
| 2016 | 80022 (16.4) | 69965 (16.3) | 10057 (16.7) |
| 2017 | 81554 (16.7) | 71533 (16.7) | 10021 (16.6) |
| 2018 | 82051 (16.8) | 72209 (16.8) | 9842 (16.3) |
| 2019 | 40711 (8.3) | 35691 (8.3) | 5020 (8.3) |
| onset_to_admission_mins (mean (SD)) | 2083.57 (188698.57) | 2226.84 (200003.14) | 986.52 (44264.70) |
| congestive_heart_failure (%) | 26317 (5.4) | 20656 (4.8) | 5661 (9.4) |
| hypertension (%) | 264806 (54.2) | 232505 (54.2) | 32301 (53.5) |
| atrial_fibrillation (%) | 96354 (19.7) | 76417 (17.8) | 19937 (33.0) |
| diabetes (%) | 102324 (20.9) | 90104 (21.0) | 12220 (20.2) |
| previous_stroke_tia (%) | 129462 (26.5) | 112358 (26.2) | 17104 (28.3) |
| prior_anticoagulation_if_Afib (%) |  |  |  |
| Yes | 52246 (26.0) | 42065 (24.6) | 10181 (33.4) |
| No | 129544 (64.4) | 112705 (66.0) | 16839 (55.3) |
| No but | 19349 (9.6) | 15895 (9.3) | 3454 (11.3) |
| inr_at_admission (median [IQR]) | 2.00 [1.50, 2.60] | 2.00 [1.50, 2.60] | 2.00 [1.40, 2.70] |
| rankin_scale_prestroke (%) |  |  |  |
| 0 | 266409 (54.5) | 245979 (57.4) | 20430 (33.8) |
| 1 | 75661 (15.5) | 66940 (15.6) | 8721 (14.4) |
| 2 | 50795 (10.4) | 42592 (9.9) | 8203 (13.6) |
| 3 | 56994 (11.7) | 44601 (10.4) | 12393 (20.5) |
| 4 | 30276 (6.2) | 22339 (5.2) | 7937 (13.1) |
| 5 | 8812 (1.8) | 6134 (1.4) | 2678 (4.4) |
| nihss_arrival (mean (SD)) | 7.09 (7.58) | 5.87 (6.25) | 15.79 (10.12) |
| nihss_loss_of_consciousness (%) |  |  |  |
| 0 | 413153 (84.5) | 387068 (90.3) | 26085 (43.2) |
| 1 | 44983 (9.2) | 30155 (7.0) | 14828 (24.6) |
| 2 | 18946 (3.9) | 8604 (2.0) | 10342 (17.1) |
| 3 | 11865 (2.4) | 2758 (0.6) | 9107 (15.1) |
| nihss_answers_questions (%) |  |  |  |
| 0 | 298837 (66.6) | 285375 (72.0) | 13462 (25.9) |
| 1 | 42868 (9.6) | 37111 (9.4) | 5757 (11.1) |
| 2 | 106826 (23.8) | 74088 (18.7) | 32738 (63.0) |
| nihss_obeys_commands (%) |  |  |  |
| 0 | 360609 (80.3) | 339614 (85.6) | 20995 (40.3) |
| 1 | 34841 (7.8) | 26863 (6.8) | 7978 (15.3) |
| 2 | 53612 (11.9) | 30484 (7.7) | 23128 (44.4) |
| nihss_best_gaze (%) |  |  |  |
| 0 | 362151 (81.4) | 339456 (86.0) | 22695 (45.2) |
| 1 | 48175 (10.8) | 36505 (9.3) | 11670 (23.2) |
| 2 | 34464 (7.7) | 18594 (4.7) | 15870 (31.6) |
| nihss_visual_deficits (%) |  |  |  |
| 0 | 324676 (73.8) | 302069 (77.1) | 22607 (46.6) |
| 1 | 46904 (10.7) | 40491 (10.3) | 6413 (13.2) |
| 2 | 60292 (13.7) | 44546 (11.4) | 15746 (32.5) |
| 3 | 8340 (1.9) | 4641 (1.2) | 3699 (7.6) |
| nihss_facial_weakness (%) |  |  |  |
| 0 | 225946 (50.3) | 211907 (53.3) | 14039 (27.2) |
| 1 | 139021 (31.0) | 122546 (30.8) | 16475 (31.9) |
| 2 | 72154 (16.1) | 55633 (14.0) | 16521 (32.0) |
| 3 | 11756 (2.6) | 7172 (1.8) | 4584 (8.9) |
| nihss_left_arm_weakness (%) |  |  |  |
| 0 | 299830 (66.7) | 276712 (69.6) | 23118 (44.6) |
| 1 | 66621 (14.8) | 62140 (15.6) | 4481 (8.6) |
| 2 | 28402 (6.3) | 23858 (6.0) | 4544 (8.8) |
| 3 | 17378 (3.9) | 12525 (3.2) | 4853 (9.4) |
| 4 | 37092 (8.3) | 22214 (5.6) | 14878 (28.7) |
| nihss_right_arm_weakness (%) |  |  |  |
| 0 | 311147 (69.2) | 287972 (72.5) | 23175 (44.7) |
| 1 | 59331 (13.2) | 55152 (13.9) | 4179 (8.1) |
| 2 | 24587 (5.5) | 20439 (5.1) | 4148 (8.0) |
| 3 | 17130 (3.8) | 12199 (3.1) | 4931 ( 9.5) |
| 4 | 37131 (8.3) | 21680 (5.5) | 15451 (29.8) |
| nihss_left_leg_weakness (%) |  |  |  |
| 0 | 301501 (67.2) | 280320 (70.6) | 21181 (41.0) |
| 1 | 56590 (12.6) | 52363 (13.2) | 4227 (8.2) |
| 2 | 35184 (7.8) | 29070 (7.3) | 6114 (11.8) |
| 3 | 24784 (5.5) | 17680 (4.5) | 7104 (13.7) |
| 4 | 30488 (6.8) | 17436 (4.4) | 13052 (25.3) |
| nihss_right_leg_weakness (%) |  |  |  |
| 0 | 312703 (69.7) | 291227 (73.4) | 21476 (41.6) |
| 1 | 48881 (10.9) | 45012 (11.3) | 3869 (7.5) |
| 2 | 30959 (6.9) | 25319 (6.4) | 5640 (10.9) |
| 3 | 23986 (5.3) | 17013 (4.3) | 6973 (13.5) |
| 4 | 31962 (7.1) | 18250 (4.6) | 13712 (26.5) |
| nihss_ataxia (%) |  |  |  |
| 0 | 348530 (79.6) | 309086 (79.3) | 39444 (82.2) |
| 1 | 61131 (14.0) | 57064 (14.6) | 4067 (8.5) |
| 2 | 27944 (6.4) | 23488 (6.0) | 4456 (9.3) |
| nihss_sensory_loss (%) |  |  |  |
| 0 | 298758 (67.9) | 276647 (70.6) | 22111 (45.5) |
| 1 | 95655 (21.7) | 85226 (21.8) | 10429 (21.5) |
| 2 | 45879 (10.4) | 29811 (7.6) | 16068 (33.1) |
| nihss_best_language (%) |  |  |  |
| 0 | 283097 (63.2) | 268244 (67.7) | 14853 (28.9) |
| 1 | 67169 (15.0) | 60897 (15.4) | 6272 (12.2) |
| 2 | 53619 (12.0) | 43492 (11.0) | 10127 (19.7) |
| 3 | 43939 (9.8) | 23817 (6.0) | 20122 (39.2) |
| nihss_dysarthria (%) |  |  |  |
| 0 | 233793 (52.5) | 220283 (55.8) | 13510 (27.1) |
| 1 | 143419 (32.2) | 130107 (32.9) | 13312 (26.7) |
| 2 | 67704 (15.2) | 44657 (11.3) | 23047 (46.2) |
| nihss_extinction (%) |  |  |  |
| 0 | 329920 (74.4) | 310098 (78.8) | 19822 (39.8) |
| 1 | 60663 (13.7) | 50136 (12.7) | 10527 (21.1) |
| 2 | 52834 (11.9) | 33396 (8.5) | 19438 (39.0) |
| type_of_stroke = Primary Intracerebral Haemorrhage (%) | 55758 (11.5) | 39472 (9.3) | 16286 (27.2) |
| intraarterial_intervention (%) | 2691 (1.0) | 2278 (0.9) | 413 (1.5) |
| systolic_blood_pressure_arrival (mean (SD)) | 167.72 (32.92) | 165.96 (31.99) | 172.11 (34.75) |
| palliative_care_within72h (%) | 25437 (5.2) | 2898 (0.7) | 22539 (37.3) |
| worst_level_of_consciousness (%) |  |  |  |
| 0 | 391065 (80.0) | 379085 (88.5) | 11980 (19.8) |
| 1 | 41916 (8.6) | 32715 (7.6) | 9201 (15.2) |
| 2 | 22635 (4.6) | 11790 (2.8) | 10845 (18.0) |
| 3 | 33331 (6.8) | 4995 (1.2) | 28336 (46.9) |
| pneumonia_requiring_antibiotics (%) |  |  |  |
| Yes | 41536 (8.5) | 25759 (6.0) | 15777 (26.1) |
| No | 442025 (90.4) | 399018 (93.1) | 43007 (71.2) |
| Not known | 5386 (1.1) | 3808 (0.9) | 1578 (2.6) |
| LOST_team_days (mean (SD)) | 16.74 (24.49) | 17.81 (25.84) | 9.13 (7.27) |
| total_IP_LOS_days (mean (SD)) | 18.37 (26.99) | 19.62 (28.47) | 9.52 (7.52) |
| discharge_destination (%) |  |  |  |
| Died | 71139 (14.5) | 10777 (2.5) | 60362 (100.0) |
| Was discharged home | 194989 (39.9) | 194989 (45.5) | 0 (0.0) |
| Was discharged to a care home | 47751 (9.8) | 47751 (11.1) | 0 (0.0) |
| Was discharged to somewhere else | 15379 (3.1) | 15379 (3.6) | 0 (0.0) |
| Was transferred to an ESD/community team | 147143 (30.1) | 147143 (34.3) | 0 (0.0) |
| Was transferred to ESD/CRT NOT PARTICIPATING | 12546 (2.6) | 12546 (2.9) | 0 (0.0) |
| rankin_scale_discharge (%) |  |  |  |
| 0 | 66765 (13.7) | 66765 (15.6) | 0 (0.0) |
| 1 | 93014 (19.0) | 93014 (21.7) | 0 (0.0) |
| 2 | 77310 (15.8) | 77310 (18.0) | 0 (0.0) |
| 3 | 81940 (16.8) | 81940 (19.1) | 0 (0.0) |
| 4 | 66757 (13.7) | 66757 (15.6) | 0 (0.0) |
| 5 | 32022 ( 6.5) | 32022 (7.5) | 0 (0.0) |
| 6 | 71139 (14.5) | 10777 (2.5) | 60362 (100.0) |
| previously_carehome_resident (%) | 17003 (35.6) | 17003 (35.6) | 0 (NaN) |
| help_activities_daily_living (%) | 165203 (39.5) | 165203 (39.5) | 0 (NaN) |
| atrial_fibrillation_discharge (%) | 91211 (21.8) | 91211 (21.8) | 0 (NaN) |
| rankin_scale_6months (mean (SD)) | 2.34 (1.86) | 2.34 (1.86) | NaN (NA) |
| clock_start_brain_scan_mins (median [IQR]) | 63.00 [25.00, 165.00] | 67.00 [26.00, 173.00] | 44.00 [22.00, 104.00] |
| direct_admission_SU_4h (%) | 272384 (59.0) | 241010 (59.1) | 31374 (57.9) |
| SU_at_least_90p (%) | 394408 (80.7) | 342863 (80.0) | 51545 (85.4) |
| received_thrombolysis (%) |  |  |  |
| Yes | 56210 (11.5) | 49469 (11.5) | 6741 (11.2) |
| No | 6626 (1.4) | 5932 (1.4) | 694 (1.1) |
| No but | 426111 (87.1) | 373184 (87.1) | 52927 (87.7) |
| thrombolysed_within_1h (%) | 33585 (59.7) | 29847 (60.3) | 3738 (55.5) |
| clock_start_thrombolysis (median [IQR]) | 53.00 [37.00, 78.00] | 53.00 [36.00, 77.00] | 56.00 [39.00, 84.00] |
| stroke_physician_24h (%) | 390839 (79.9) | 344833 (80.5) | 46006 (76.2) |
| clock_start_stroke_physician (median [IQR]) | 696.00 [120.00, 1214.00] | 716.00 [124.00, 1219.00] | 520.00 [92.00, 1161.00] |
| stroke_nurse_24h (%) | 435642 (89.1) | 383857 (89.6) | 51785 (85.8) |
| clock_start_stroke_nurse (median [IQR]) | 84.00 [7.00, 266.00] | 86.00 [8.00, 267.00] | 73.00 [4.00, 257.00] |
| swallow_screen_within_4h (%) | 316949 (71.1) | 284638 (71.0) | 32311 (72.5) |
| swallow_assessment_within_72h (%) | 160410 (84.7) | 136991 (83.5) | 23419 (92.4) |
| required_occupational_therapy (%) | 403280 (82.5) | 376288 (87.8) | 26992 (44.7) |
| percentage_ot_days (median [IQR]) | 50.00 [33.33, 83.33] | 50.00 [33.33, 84.15] | 42.86 [25.00, 66.67] |
| assessment_ot_within_72h (%) | 381647 (90.7) | 351961 (90.6) | 29686 (93.0) |
| required_physiotherapy (%) | 413179 (84.5) | 378866 (88.4) | 34313 (56.8) |
| percentage_pt_days (mean (SD)) | 61.83 (28.27) | 62.25 (28.24) | 57.27 (28.28) |
| assessment_pt_within_72h (%) | 410444 (94.5) | 374208 (94.3) | 36236 (96.0) |
| required_speech_therapy (%) | 230328 (47.1) | 205112 (47.9) | 25216 (41.8) |
| percentage_salt_days (median [IQR]) | 37.50 [22.22, 60.00] | 37.21 [21.62, 60.00] | 40.00 [25.00, 60.00] |
| assessment_salt_within_72h (%) | 199020 (85.8) | 177675 (85.3) | 21345 (90.5) |
| early_supported_discharge (%) |  |  |  |
| Yes, stroke/neurology specific | 143475 (34.3) | 143475 (34.3) | 0 (NaN) |
| Yes, non-specialist | 4084 (1.0) | 4084 (1.0) | 0 (NaN) |
| No | 270249 (64.7) | 270249 (64.7) | 0 (NaN) |

Table D: Participant Characteristics for development, validation and temporal validation set

|  | **Whole dataset for predicting 30-day mortality** | | |
| --- | --- | --- | --- |
|  | **2013-2018 training** | **2013-2018 validation** | **2019 temporal validation** |
| n | 358588 | 89649 | 40711 |
| Age_Groups_by5 (%) |  |  |  |
| (15,20] | 365 (0.1) | 100 (0.1) | 29 (0.1) |
| (20,25] | 583 (0.2) | 113 (0.1) | 57 (0.1) |
| (25,30] | 926 (0.3) | 251 (0.3) | 105 (0.3) |
| (30,35] | 1551 (0.4) | 411 (0.5) | 182 (0.4) |
| (35,40] | 2691 (0.8) | 646 (0.7) | 339 (0.8) |
| (40,45] | 5503 (1.5) | 1327 (1.5) | 554 (1.4) |
| (45,50] | 10128 (2.8) | 2401 (2.7) | 1104 (2.7) |
| (50,55] | 15125 (4.2) | 3808 (4.2) | 1745 (4.3) |
| (55,60] | 19605 (5.5) | 4876 (5.4) | 2416 (5.9) |
| (60,65] | 25918 (7.2) | 6484 (7.2) | 3050 (7.5) |
| (65,70] | 36153 (10.1) | 9005 (10.0) | 3874 (9.5) |
| (70,75] | 45053 (12.6) | 11357 (12.7) | 5423 (13.3) |
| (75,80] | 54984 (15.3) | 13774 (15.4) | 6137 (15.1) |
| (80,85] | 59835 (16.7) | 14951 (16.7) | 6727 (16.5) |
| (85,90] | 48425 (13.5) | 12147 (13.5) | 5497 (13.5) |
| (90,95] | 25417 (7.1) | 6409 (7.1) | 2700 (6.6) |
| (95,100] | 5730 (1.6) | 1446 (1.6) | 714 (1.8) |
| (100,105] | 569 (0.2) | 136 (0.2) | 53 (0.1) |
| (105,110] | 10 (0.0) | 3 (0.0) | 2 (0.0) |
| (110,115] | 7 (0.0) | 0 (0.0) | 2 (0.0) |
| (115,120] | 10 (0.0) | 3 (0.0) | 1 (0.0) |
| Male (%) | 182457 (50.9) | 45644 (50.9) | 21190 (52.0) |
| Ethnicity (%) |  |  |  |
| White | 317872 (88.6) | 79412 (88.6) | 35627 (87.5) |
| Black | 4702 (1.3) | 1200 (1.3) | 574 (1.4) |
| Asian | 10492 (2.9) | 2703 (3.0) | 1352 (3.3) |
| Mixed | 1311 (0.4) | 316 (0.4) | 188 (0.5) |
| Other | 4134 (1.2) | 1030 (1.1) | 581 (1.4) |
| Unknown | 20077 (5.6) | 4987 (5.6) | 2389 (5.9) |
| Inpatient_at_time_of_stroke (%) | 19808 (5.5) | 5001 (5.6) | 2291 (5.6) |
| hour_of_admission_4h_band (%) |  |  |  |
| 00:00:00 to 03:59:59 | 18332 (5.1) | 4607 (5.1) | 2178 (5.3) |
| 04:00:00 to 07:59:59 | 19875 (5.5) | 4866 (5.4) | 2092 (5.1) |
| 08:00:00 to 11:59:59 | 98509 (27.5) | 24611 (27.5) | 10766 (26.4) |
| 12:00:00 to 15:59:59 | 99687 (27.8) | 24968 (27.9) | 11706 (28.8) |
| 16:00:00 to 19:59:59 | 74868 (20.9) | 18796 (21.0) | 8601 (21.1) |
| 20:00:00 to 23:59:59 | 47317 (13.2) | 11800 (13.2) | 5368 (13.2) |
| day_of_week_of_admission (%) |  |  |  |
| Monday | 56393 (15.7) | 14038 (15.7) | 6077 (14.9) |
| Tuesday | 53644 (15.0) | 13385 (14.9) | 6043 (14.8) |
| Wednesday | 53166 (14.8) | 13247 (14.8) | 6114 (15.0) |
| Thursday | 52157 (14.5) | 13247 (14.8) | 6080 (14.9) |
| Friday | 51799 (14.4) | 13098 (14.6) | 6043 (14.8) |
| Saturday | 45530 (12.7) | 11248 (12.5) | 5191 (12.8) |
| Sunday | 45899 (12.8) | 11385 (12.7) | 5163 (12.7) |
| onset_to_admission_mins (mean (SD)) | 1983.79 (154842.60) | 3120.84 (314260.79) | 716.86 (3746.49) |
| congestive_heart_failure (%) | 19284 (5.4) | 4961 (5.5) | 2072 (5.1) |
| hypertension (%) | 193678 (54.0) | 48730 (54.4) | 22398 (55.0) |
| atrial_fibrillation = Yes (%) | 70773 (19.7) | 17791 (19.8) | 7790 (19.1) |
| diabetes = Yes (%) | 74593 (20.8) | 18783 (21.0) | 8948 (22.0) |
| previous_stroke_tia = Yes (%) | 94998 (26.5) | 23864 (26.6) | 10600 (26.0) |
| prior_anticoagulation_if_Afib (%) |  |  |  |
| Yes | 37010 (28.9) | 9175 (28.5) | 6061 (14.9) |
| No | 78246 (61.0) | 19667 (61.2) | 31631 (77.7) |
| No but | 13017 (10.1) | 3313 (10.3) | 3019 (7.4) |
| inr_at_admission (median [IQR]) | 2.00 [1.50, 2.70] | 1.90 [1.50, 2.50] | 2.00 [1.50, 2.50] |
| rankin_scale_prestroke (%) |  |  |  |
| 0 | 196470 (54.8) | 48835 (54.5) | 21104 (51.8) |
| 1 | 54819 (15.3) | 13870 (15.5) | 6972 (17.1) |
| 2 | 36980 (10.3) | 9317 (10.4) | 4498 (11.0) |
| 3 | 41625 (11.6) | 10440 (11.6) | 4929 (12.1) |
| 4 | 22206 (6.2) | 5553 (6.2) | 2517 (6.2) |
| 5 | 6488 (1.8) | 1633 (1.8) | 691 (1.7) |
| nihss_arrival (mean (SD)) | 7.08 (7.57) | 7.09 (7.60) | 7.19 (7.69) |
| nihss_loss_of_consciousness (%) |  |  |  |
| 0 | 302387 (84.3) | 75855 (84.6) | 34911 (85.8) |
| 1 | 33463 (9.3) | 8084 (9.0) | 3436 (8.4) |
| 2 | 13976 (3.9) | 3541 (3.9) | 1429 (3.5) |
| 3 | 8762 (2.4) | 2168 (2.4) | 935 (2.3) |
| nihss_answers_questions (%) |  |  |  |
| 0 | 217851 (66.6) | 54608 (66.6) | 26378 (67.1) |
| 1 | 31176 (9.5) | 7849 (9.6) | 3843 (9.8) |
| 2 | 78161 (23.9) | 19581 (23.9) | 9084 (23.1) |
| nihss_obeys_commands (%) |  |  |  |
| 0 | 262937 (80.3) | 65746 (80.1) | 31926 (81.1) |
| 1 | 25466 (7.8) | 6455 (7.9) | 2920 (7.4) |
| 2 | 39190 (12.0) | 9926 (12.1) | 4496 (11.4) |
| nihss_best_gaze (%) |  |  |  |
| 0 | 263659 (81.3) | 66234 (81.5) | 32258 (82.3) |
| 1 | 35490 (10.9) | 8772 (10.8) | 3913 (10.0) |
| 2 | 25193 (7.8) | 6270 (7.7) | 3001 (7.7) |
| nihss_visual_deficits (%) |  |  |  |
| 0 | 236405 (73.7) | 59190 (73.6) | 29081 (74.7) |
| 1 | 34323 (10.7) | 8697 (10.8) | 3884 (10.0) |
| 2 | 44111 (13.7) | 10959 (13.6) | 5222 (13.4) |
| 3 | 6023 (1.9) | 1562 (1.9) | 755 (1.9) |
| nihss_facial_weakness (%) |  |  |  |
| 0 | 163997 (50.1) | 41099 (50.1) | 20850 (53.1) |
| 1 | 101580 (31.0) | 25605 (31.2) | 11836 (30.1) |
| 2 | 53266 (16.3) | 13164 (16.0) | 5724 (14.6) |
| 3 | 8646 (2.6) | 2238 (2.7) | 872 (2.2) |
| nihss_left_arm_weakness (%) |  |  |  |
| 0 | 218243 (66.6) | 54780 (66.6) | 26807 (68.1) |
| 1 | 48604 (14.8) | 12152 (14.8) | 5865 (14.9) |
| 2 | 20931 (6.4) | 5191 (6.3) | 2280 (5.8) |
| 3 | 12733 (3.9) | 3230 (3.9) | 1415 (3.6) |
| 4 | 27259 (8.3) | 6838 (8.3) | 2995 (7.6) |
| nihss_right_arm_weakness (%) |  |  |  |
| 0 | 226516 (69.1) | 56809 (69.1) | 27822 (70.7) |
| 1 | 43321 (13.2) | 10945 (13.3) | 5065 (12.9) |
| 2 | 18036 (5.5) | 4593 (5.6) | 1958 (5.0) |
| 3 | 12640 (3.9) | 3087 (3.8) | 1403 (3.6) |
| 4 | 27249 (8.3) | 6769 (8.2) | 3113 (7.9) |
| nihss_left_leg_weakness (%) |  |  |  |
| 0 | 219604 (67.1) | 55120 (67.2) | 26777 (68.1) |
| 1 | 41430 (12.7) | 10238 (12.5) | 4922 (12.5) |
| 2 | 25845 (7.9) | 6388 (7.8) | 2951 (7.5) |
| 3 | 18075 (5.5) | 4626 (5.6) | 2083 (5.3) |
| 4 | 22218 (6.8) | 5682 (6.9) | 2588 (6.6) |
| nihss_right_leg_weakness (%) |  |  |  |
| 0 | 227906 (69.7) | 57203 (69.7) | 27594 (70.2) |
| 1 | 35620 (10.9) | 9018 (11.0) | 4243 (10.8) |
| 2 | 22616 (6.9) | 5687 (6.9) | 2656 (6.8) |
| 3 | 17563 (5.4) | 4327 (5.3) | 2096 (5.3) |
| 4 | 23422 (7.2) | 5816 (7.1) | 2724 (6.9) |
| nihss_ataxia (%) |  |  |  |
| 0 | 254281 (79.8) | 63754 (79.7) | 30495 (78.6) |
| 1 | 44319 (13.9) | 11163 (14.0) | 5649 (14.6) |
| 2 | 20244 (6.3) | 5058 (6.3) | 2642 (6.8) |
| nihss_sensory_loss (%) |  |  |  |
| 0 | 217479 (67.8) | 54685 (68.0) | 26594 (68.4) |
| 1 | 69872 (21.8) | 17386 (21.6) | 8397 (21.6) |
| 2 | 33646 (10.5) | 8348 (10.4) | 3885 (10.0) |
| nihss_best_language (%) |  |  |  |
| 0 | 205986 (63.0) | 51746 (63.2) | 25365 (64.7) |
| 1 | 49204 (15.1) | 12152 (14.8) | 5813 (14.8) |
| 2 | 39371 (12.1) | 9927 (12.1) | 4321 (11.0) |
| 3 | 32159 (9.8) | 8060 (9.8) | 3720 (9.5) |
| nihss_dysarthria (%) |  |  |  |
| 0 | 169896 (52.4) | 42967 (52.8) | 20930 (53.5) |
| 1 | 105130 (32.4) | 25986 (31.9) | 12303 (31.5) |
| 2 | 49445 (15.2) | 12389 (15.2) | 5870 (15.0) |
| nihss_extinction (%) |  |  |  |
| 0 | 240336 (74.3) | 60225 (74.3) | 29359 (75.2) |
| 1 | 44392 (13.7) | 11137 (13.7) | 5134 (13.2) |
| 2 | 38630 (11.9) | 9669 (11.9) | 4535 (11.6) |
| type_of_stroke = Primary Intracerebral Haemorrhage (%) | 40681 (11.4) | 10175 (11.4) | 4902 (12.1) |
| Intra arterial_intervention (%) | 1711 (0.9) | 424 (0.9) | 556 (1.6) |
| systolic_blood_pressure_arrival (mean (SD)) | 167.67 (33.02) | 167.49 (33.47) | 167.89 (32.52) |
| palliative_care_within72h (%) | 18618 (5.2) | 4546 (5.1) | 2273 (5.6) |
| worst_level_of_consciousness (%) |  |  |  |
| 0 | 286051 (79.8) | 71677 (80.0) | 33337 (81.9) |
| 1 | 31101 (8.7) | 7690 (8.6) | 3125 (7.7) |
| 2 | 16848 (4.7) | 4149 (4.6) | 1638 (4.0) |
| 3 | 24588 (6.9) | 6132 (6.8) | 2611 (6.4) |
| pneumonia_requiring_antibiotics (%) |  |  |  |
| Yes | 30670 (8.6) | 7654 (8.5) | 3212 (7.9) |
| No | 323840 (90.3) | 81024 (90.4) | 37161 (91.3) |
| Not known | 4078 (1.1) | 970 (1.1) | 338 (0.8) |
| LOST_team_days (mean (SD)) | 16.93 (24.84) | 16.86 (24.78) | 14.83 (20.33) |
| total_IP_LOS_days (mean (SD)) | 18.57 (27.37) | 18.50 (27.28) | 16.33 (22.50) |
| discharge_destination (%) |  |  |  |
| Died | 52451 (14.6) | 12901 (14.4) | 5787 (14.2) |
| Was discharged home | 145549 (40.6) | 36549 (40.8) | 12891 (31.7) |
| Was discharged to a care home | 35527 (9.9) | 8961 (10.0) | 3263 (8.0) |
| Was discharged to somewhere else | 11758 (3.3) | 2977 (3.3) | 644 (1.6) |
| Was transferred to an ESD/community team | 104202 (29.1) | 25986 (29.0) | 16955 (41.6) |
| Was transferred to ESD/CRT NOT PARTICIPATING | 9101 (2.5) | 2274 (2.5) | 1171 (2.9) |
| rankin_scale_discharge (%) |  |  |  |
| 0 | 49774 (13.9) | 12374 (13.8) | 4617 (11.3) |
| 1 | 68223 (19.0) | 17144 (19.1) | 7647 (18.8) |
| 2 | 55754 (15.5) | 13991 (15.6) | 7565 (18.6) |
| 3 | 59704 (16.6) | 14983 (16.7) | 7253 (17.8) |
| 4 | 48996 (13.7) | 12280 (13.7) | 5481 (13.5) |
| 5 | 23686 (6.6) | 5975 (6.7) | 2361 (5.8) |
| 6 | 52451 (14.6) | 12901 (14.4) | 5787 (14.2) |
| previously_carehome_resident (%) | 12498 (35.2) | 3250 (36.3) | 1255 (38.5) |
| help_activities_daily_living (%) | 121572 (39.7) | 30378 (39.6) | 13253 (37.9) |
| atrial_fibrillation_discharge (%) | 67145 (21.9) | 16707 (21.8) | 7359 (21.1) |
| rankin_scale_6months (mean (SD)) | 2.34 (1.85) | 2.31 (1.84) | 2.39 (1.96) |
| clock_start_brain_scan_mins (median [IQR]) | 64.00 [26.00, 167.00] | 65.00 [26.00, 169.00] | 52.00 [22.00, 140.00] |
| direct_admission_SU_4h (%) | 200329 (59.1) | 50193 (59.3) | 21862 (56.9) |
| SU_at_least_90p (%) | 289783 (80.8) | 72177 (80.5) | 32448 (79.7) |
| received_thrombolysis (%) |  |  |  |
| Yes | 41278 (11.5) | 10238 (11.4) | 4694 (11.5) |
| No | 5035 (1.4) | 1261 (1.4) | 330 (0.8) |
| No but | 312275 (87.1) | 78149 (87.2) | 35687 (87.7) |
| thrombolysed_within_1h (%) | 24659 (59.7) | 6047 (59.1) | 2879 (61.3) |
| clock_start_thrombolysis (median [IQR]) | 53.00 [37.00, 78.00] | 54.00 [37.00, 79.00] | 53.00 [36.00, 76.00] |
| stroke_physician_24h (%) | 285335 (79.6) | 71239 (79.5) | 34265 (84.2) |
| clock_start_stroke_physician (median [IQR]) | 706.00 [120.00, 1222.00] | 708.00 [121.00, 1223.00] | 580.00 [106.00, 1110.00] |
| stroke_nurse_24h (%) | 318966 (89.0) | 79662 (88.9) | 37014 (90.9) |
| clock_start_stroke_nurse (median [IQR]) | 87.00 [8.00, 268.00] | 87.00 [8.00, 266.00] | 60.00 [3.00, 243.00] |
| swallow_screen_within_4h (%) | 230954 (70.8) | 57719 (70.9) | 28276 (74.3) |
| swallow_assessment_within_72h (%) | 118269 (84.5) | 29452 (84.1) | 12689 (87.8) |
| required_occupational_therapy (%) | 295154 (82.3) | 73793 (82.3) | 34333 (84.3) |
| percentage_ot_days (median [IQR]) | 50.00 [33.33, 80.00] | 50.00 [33.33, 80.00] | 50.00 [33.33, 100.00] |
| assessment_ot_within_72h (%) | 278591 (90.5) | 69600 (90.4) | 33456 (93.5) |
| required_physiotherapy (%) | 302910 (84.5) | 75941 (84.7) | 34328 (84.3) |
| percentage_pt_days (mean (SD)) | 61.63 (28.27) | 61.71 (28.36) | 63.64 (28.05) |
| assessment_pt_within_72h (%) | 300625 (94.4) | 75255 (94.4) | 34564 (95.3) |
| required_speech_therapy (%) | 168777 (47.1) | 42172 (47.0) | 19379 (47.6) |
| percentage_salt_days (median [IQR]) | 36.36 [21.54, 60.00] | 36.84 [21.43, 58.61] | 42.86 [25.00, 66.67] |
| assessment_salt_within_72h (%) | 144722 (85.5) | 36142 (85.3) | 18156 (89.3) |
| early_supported_discharge (%) |  |  |  |
| Yes, stroke/neurology specific | 103188 (33.7) | 25824 (33.6) | 14463 (41.4) |
| Yes, non-specialist | 3125 (1.0) | 738 (1.0) | 221 (0.6) |
| No | 199824 (65.3) | 50185 (65.4) | 20240 (58.0) |
| mortality_30_day (%) | 44431 (12.4) | 10911 (12.2) | 5020 (12.3) |

Table E, Brier score, AUC, calibration-in-the-large and calibration slope with 95% Confidence Interval (CI) for validation set.

| Model | Brier score (95% CI) | AUC (95% CI) | Calibration-in-the-large (95% CI) | Calibration slope (95% CI) |
| --- | --- | --- | --- | --- |
| LR reference model | 0.083 (0.081-0.084 | 0.827 (0.823-0.832) | -0.105 (-0.139--0.070) | 0.887 (0.871-0.905) |
| LR | 0.077 (0.075-0.078) | 0.873 (0.870 to 0.877) | 0.060 (0.022-0.097) | 0.986 (0.969-1.004) |
| LR with elastic net | 0.077 (0.075-0.078) | 0.874 (0.870-0.877) | 0.087 (0.048-0.124) | 1.003 (0.986- 1.022) |
| LR with elastic net and interaction terms | 0.074 (0.073-0.075) | 0.879 (0.876-0.883) | 0.085 (0.049-0.119) | 1.108 (1.002-1.035) |
| XGBoost | 0.072 (0.071-0.073) | 0.885 (0.882-0.889) | 0.048 (0.016-0.080) | 1.018 (1.001-1.036) |

Table F, Shift table of reclassification of all 30 variable models compared to LR reference model with 4 variables

|  | LR reference model (4 variables) | | | | | | | |
| --- | --- | --- | --- | --- | --- | --- | --- | --- |
|  | Low | | Moderate | | High | | All | |
|  | Number (% in Low) | observed mortality (%) | Number (% in Moderate) | observed mortality (%) | Number (% in High) | observed mortality (%) | Number (% in All) | observed mortality (%) |
| LR with 30 variables | | | | | | | |  |
| Number in Low | 19984 (93.8) | 1.64 | 2980 (27.2) | 3.56 | 10 (0.1) | 0 | 22974 (56.4) | 1.89 |
| Number in Moderate | 1213 (5.7) | 12.94 | 7024 (64.2) | 8.41 | 1445 (17.1) | 14.33 | 9682 (23.8) | 9.86 |
| Number in High | 115 (0.5) | 61.74 | 942 (8.6) | 33.44 | 6998 (82.8) | 46.37 | 8055 (19.8) | 45.08 |
| All | 21312 (100) | 2.61 | 10946 (100) | 9.25 | 8453 (100) | 40.84 | 40711 (100) | 12.33 |
| LR with elastic net with 30 variables | | | | | | | |  |
| Number in Low | 19883 (93.3) | 1.61 | 2576 (23.5) | 3.34 | 6 (0.07) | 0 | 22465 (55.2) | 1.81 |
| Number in Moderate | 1312 (6.2) | 12.42 | 7432 (67.9) | 8.21 | 1353 (16.0) | 13.75 | 10097 (24.8) | 9.50 |
| Number in High | 117 (0.5) | 62.39 | 938 (8.6) | 33.69 | 7094 (83.9) | 46.04 | 8149 (20.0) | 44.85 |
| All | 21312 (100) | 2.61 | 10946 (100) | 9.25 | 8453 (100) | 40.84 | 40711 (100) | 12.33 |
| LR with elastic net and interaction terms with 30 variables | | | | | | | |  |
| Number in Low | 19930 (93.5) | 1.57 | 3619 (33.1) | 3.43 | 6 (0.07) | 0 | 23555 (57.9) | 1.85 |
| Number in Moderate | 1175 (5.5) | 10.61 | 6199 (56.6) | 8.57 | 1364 (16.1) | 12.97 | 8741 (21.5) | 9.39 |
| Number in High | 204 (0.9) | 58.33 | 1128 (16.1) | 31.65 | 7083 (83.8) | 46.41 | 8145 (20.7) | 44.72 |
| All | 21312 (100) | 2.61 | 10946 (100) | 9.25 | 8453 (100) | 40.84 | 40711 (100) | 12.33 |
| XGBoost with 30 variables | | | | | | | |  |
| Number in Low | 19664 (92.3) | 1.45 | 3549 (32.4) | 3.07 | 6 (0.07) | 33.33 | 23219 (57.0) | 1.71 |
| Number in Moderate | 1351 (6.7) | 8.66 | 5942 (54.3) | 8.16 | 1007 (11.9) | 10.43 | 8300 (20.4) | 8.52 |
| Number in High | 297 (1.4) | 51.52 | 1455 (12.3) | 28.73 | 7440 (88.0) | 44.96 | 9192 (22.6) | 42.60 |
| All | 21312 (100) | 2.61 | 10946 (100) | 9.25 | 8453 (100) | 40.84 | 40711 (100) | 12.33 |

Note: Low is defined as risk<5%, Moderate as 5%<risk<15%, high as risk>15%

Table G, Shift table of reclassification of 30 variable models compared to LR model with 30 variables

|  | LR with 30 variables | | | | | | | |
| --- | --- | --- | --- | --- | --- | --- | --- | --- |
|  | Low | | Moderate | | High | | All | |
|  | Number (% in Low) | observed mortality (%) | Number (% in Moderate) | observed mortality (%) | Number (% in High) | observed mortality (%) | Number (% in All) | observed mortality (%) |
| LR with elastic net with 30 variables | | | | | | | |  |
| Number in Low | 22431 (97.6) | 1.80 | 34 (0.4) | 5.88 | 0 (0) | 0 | 22465 (55.2) | 1.81 |
| Number in Moderate | 543 (2.4) | 5.52 | 9491 (98) | 9.71 | 63 (0.8) | 11.11 | 10097 (24.8) | 9.50 |
| Number in High | 0 (0) | 0 | 157 (1.6) | 19.75 | 7992 (99.2) | 45.35 | 8149 (20) | 44.85 |
| All | 22974 (100) | 1.18 | 9682 (100) | 9.50 | 22974 (100) | 44.85 | 40711 (100) | 12.33 |
| LR with elastic net and interaction terms with 30 variables | | | | | | | |  |
| Number in Low | 22269 (96.9) | 1.75 | 1286 (13.3) | 3.65 | 0 (0) | 0 | 23555 (57.9) | 1.85 |
| Number in Moderate | 705 (3.1) | 6.38 | 7727 (79.8) | 9.63 | 309 (3.8) | 10.36 | 8741 (21.5) | 9.39 |
| Number in High | 0 (0) | 0 | 669 (6.9) | 24.51 | 7746 (96.2) | 46.46 | 8415 (20.7) | 44.72 |
| All | 22974 (100) | 1.18 | 9682 (100) | 9.50 | 22974 (100) | 44.85 | 40711 (100) | 12.33 |
| XGBoost with 30 variables | | | | | | | |  |
| Number in Low | 21848 (95.1) | 1.60 | 1371 (4.2) | 3.43 | 0 (0) | 0 | 23219 (57.0) | 1.71 |
| Number in Moderate | 1112 (4.8) | 7.19 | 7009 (72.4) | 8.72 | 179 (2.2) | 8.94 | 8300 (20.4) | 8.52 |
| Number in High | 14 (0.1) | 28.57 | 1302 (13.4) | 22.81 | 7876 (97.8) | 45.90 | 9192 (22.6) | 42.60 |
| All | 22974 (100) | 1.18 | 9682 (100) | 9.50 | 22974 (100) | 44.85 | 40711 (100) | 12.33 |

Note: Low is defined as risk<5%, Moderate as 5%<risk<15%, high as risk>15%

**
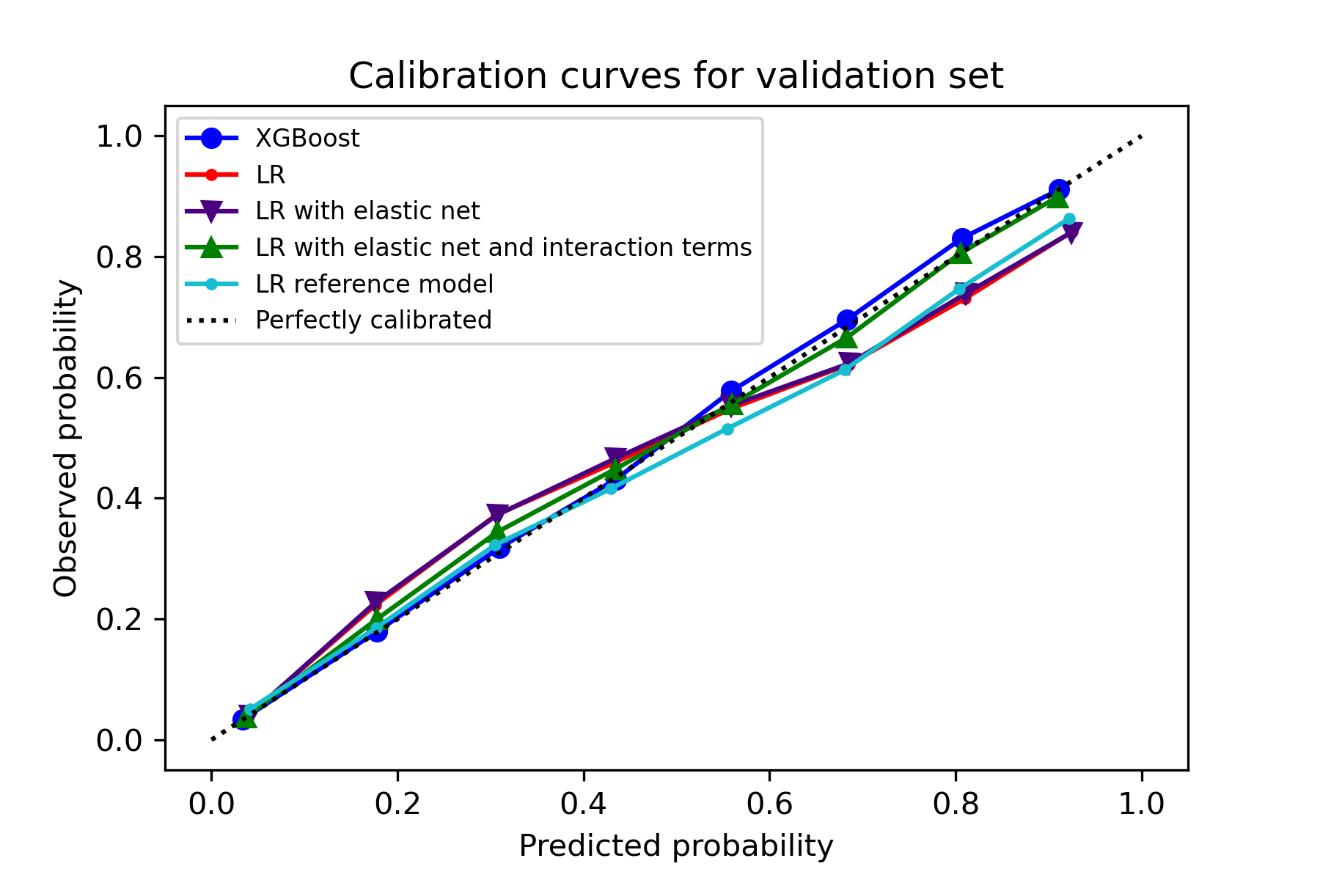
**

**Figure A: Calibration plots of LR (Reference model), LR model, LR with Elastic Net, LR with Elastic Net and interaction terms, and XGBoost.**

**
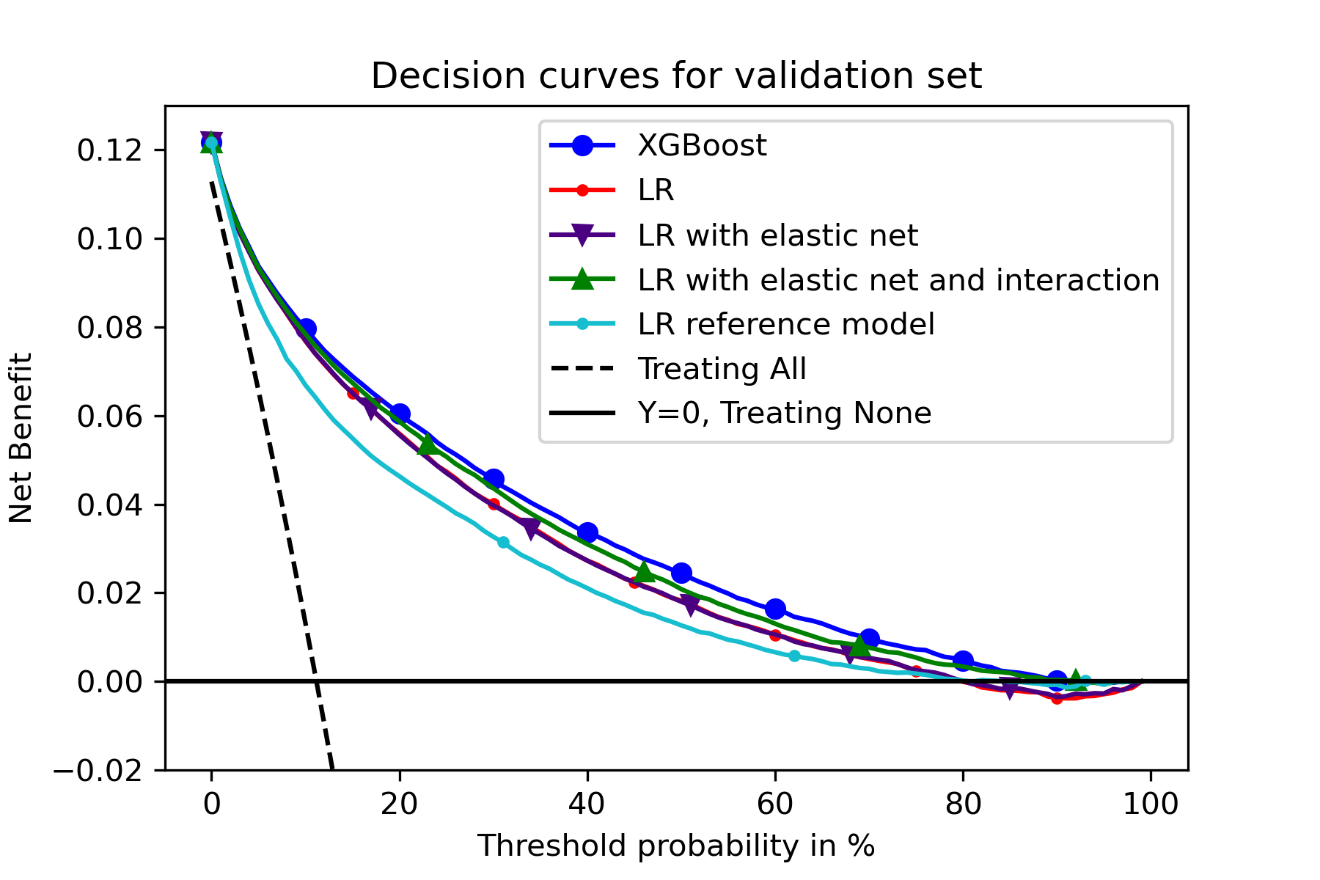
**

**Figure B: Decision curves of all models on validation set (F) for the net benefit of treated (prognosis) in function of the threshold probability.**

**
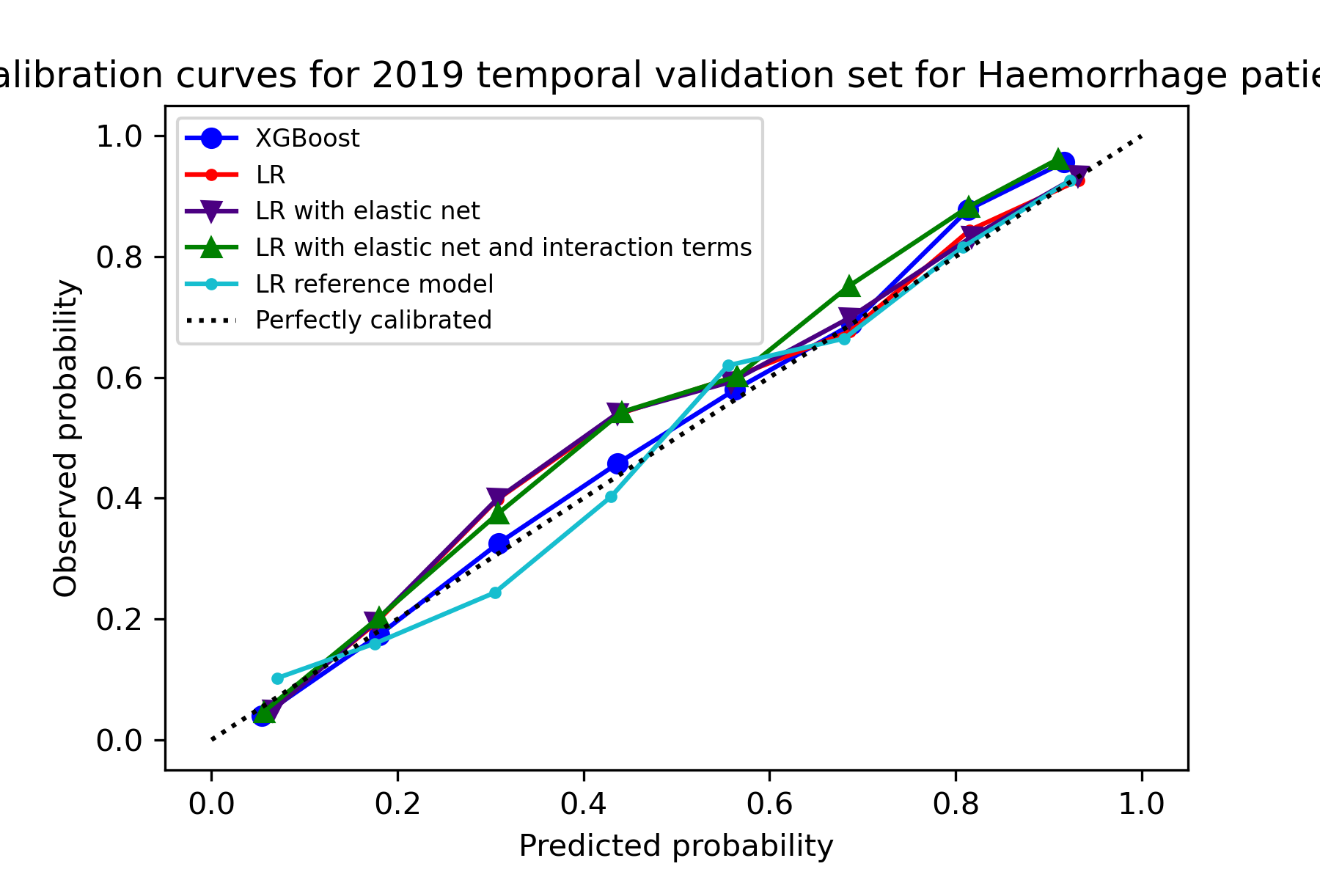
**

**Figure C: Calibration curves for all models on 2019 temporal validation set for Haemorrhage patients**

**
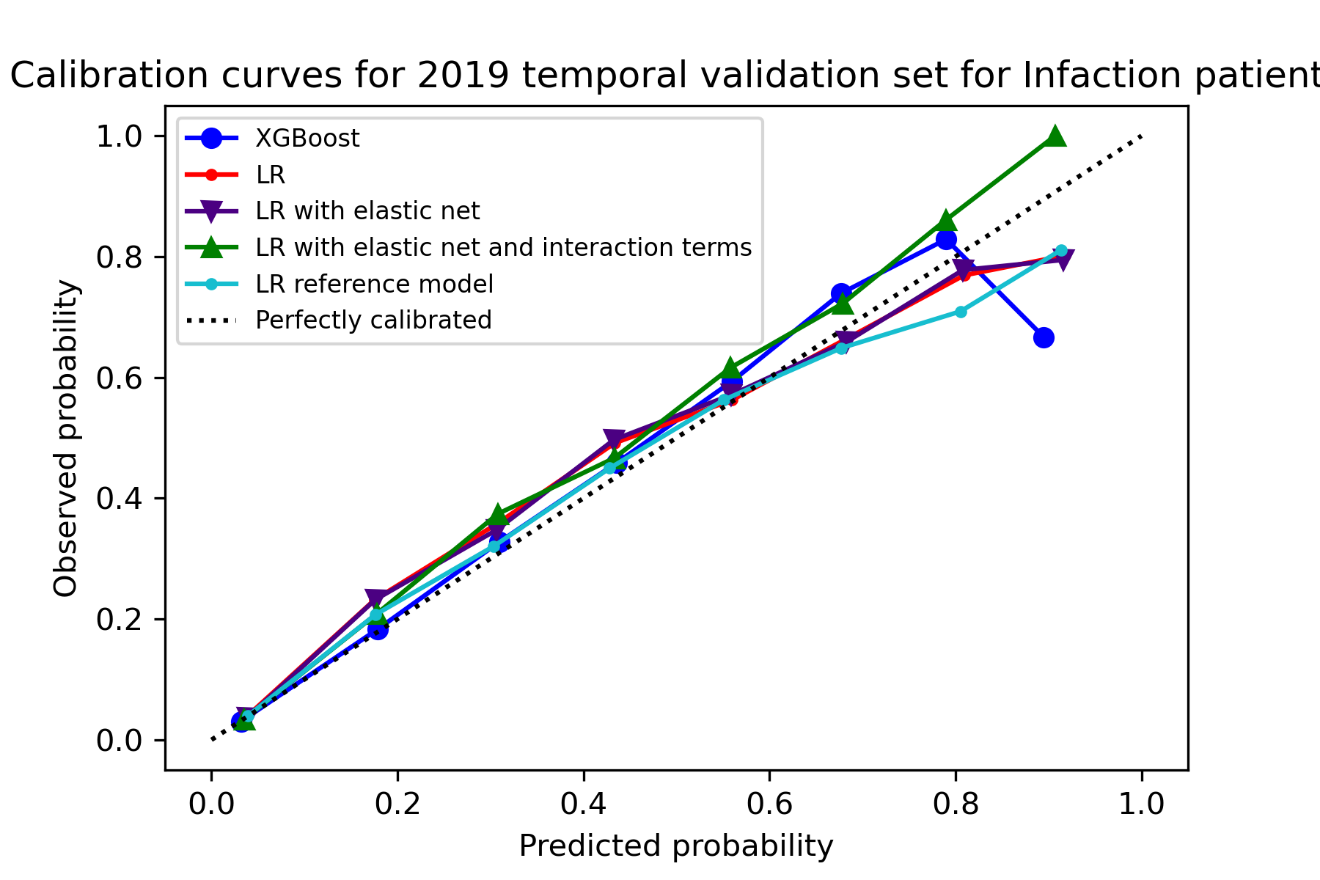
**

**Figure D: Calibration curves for all models on 2019 temporal validation set for Infarction patients**


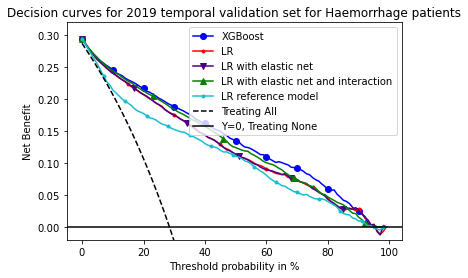


**Figure E:** Decision curves of all models for haemorrhage patients on 2019 temporal validation set

**
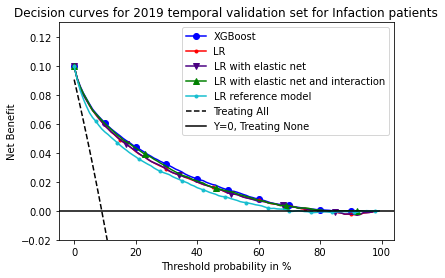
**

**Figure F:** Decision curves of all models for haemorrhage patients on 2019 temporal validation set


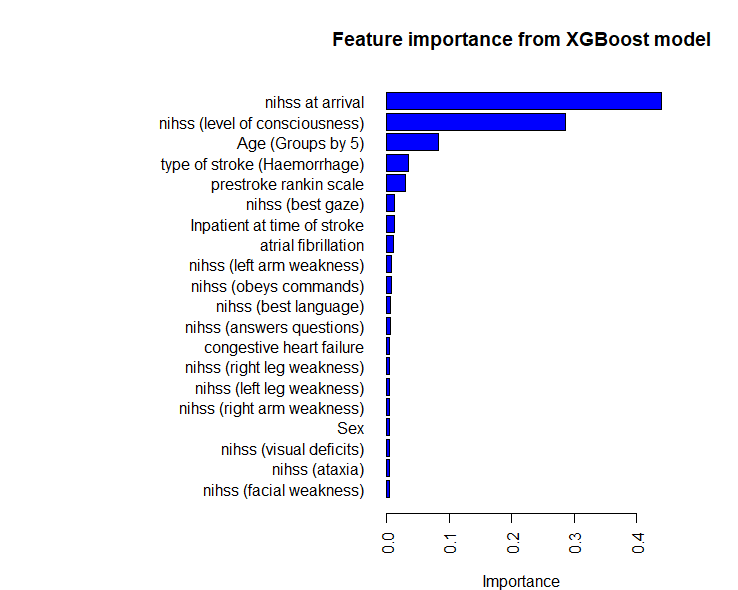


Figure G: Feature importance calculate from XGBoost model. The value implies the relative contribution of the corresponding feature to the model calculated by taking each feature's contribution for each tree in the model.
